# Time Trend of Persistent Organic Pollutants and Metals in Greenlandic Inuit during 1994–2015

**DOI:** 10.3390/ijerph18052774

**Published:** 2021-03-09

**Authors:** Manhai Long, Maria Wielsøe, Eva Cecilie Bonefeld-Jørgensen

**Affiliations:** 1 Center for Arctic Health and Molecular Epidemiology, Department of Public Health, Aarhus University, 8000 Aarhus C, Denmark; mwielsoe@ph.au.dk (M.W.); ebj@ph.au.dk (E.C.B.-J.); 2 Greenland Center for Health Research, University of Greenland, 3905 Nuuk, Greenland

**Keywords:** Greenland, arctic, biomonitoring, temporal time trend, persistent organic pollutants, heavy metals, perfluoroalkylated substances

## Abstract

Persistent organic pollutants (POPs), including polychlorinated biphenyls (PCBs), organchlorine pesticides and perfluoroalkylated substances (PFASs) and heavy metals bioaccumulate in the marine food chain in the Arctic regions, and thus, the Greenlandic population has a higher body burden due to relatively high intake of marine mammals. We assessed the temporal trend for POPs, including PCB 153; 1,1-dichloro−2,2-bis (p-chlorophenyl)-ethylene (p,p’-DDE); oxychlordane; six PFASs; mercury; lead and selenium in Inuit from Ilulissat, Nuuk, and across Greenland (including thirteen towns/districts), from 1994 to 2015. Data showed a significant annual decrease of 6.85–8.61% for PCB153, 6.67–8.61% for p,p’-DDE, 6.11–9.52% for oxychlordane, 5.92–6.76% for mercury and 6.48–9.43% for lead in Inuit women from Nuuk, Ilulissat, and across thirteen Greenlandic districts. The blood selenium level of all Greenlandic women increased 1.01% annually, while the trend direction was negative for Nuuk women. A similar pattern was seen for men across Greenland, with a yearly decrease of 11.3% for PCB 153, 8.61% for p,p’-DDE, 15.6% for oxychlordane, 13.1% for mercury and 12.2% for lead. Perfluorooctane sulfonate, perfluorohexane sulfonate and perfluorooctanoic acid significantly decreased 5.82–11.7% annually for both women and men across Greenland. For perfluorononanoic acid, perfluorodecanoic acid and perfluoroundecanoic acid, we observed an increasing trend for women across Greenland. In conclusion, there was a decreasing trend of the regulated POPs and metals but a potential increasing trend of the nonregulated PFASs in the Greenlandic population between 1994 and 2015. The continuing biomonitoring of contaminants of concern is important to protect the Arctic population heath.

## 1. Introduction

Legacy and emerging persistent organic pollutants (POPs) include lipophilic POPs, such as polychlorinated biphenyls (PCBs) and organochlorine pesticides (OCPs) and the amphiphilic perfluoroalkylated substances (PFASs). These POPs and heavy metals, such as mercury (Hg) and lead (Pb), come from long-range atmospheric transport and ocean currents released into the environment at midlatitudes and transported to the Arctic [1,2,3,4]. The legacy POPs have been of environmental concern for several decades and listed on the Stockholm Convention [5]. The legacy POPs include the PCB congeners (e.g., PCB153, PCB118, PCB 180 and dioxin-like PCBs), OCPs (e.g., aldrin; hexachlorobenzene; oxychlordane; endrin; heptachlor; mirex; toxaphene; 2,2-bis (p-chlorophenyl)−1,1,1-trichloroethane (p,p’-DDT) and its metabolite 1,1-dichloro−2,2-bis (p-chlorophenyl)-ethylene (p,p’-DDE)) and polychlorinated dibenzo-p-dioxins/furans (PCDD/PCDF) and are internationally regulated by the Stockholm Convention [5]. Due to their chemical and physical properties, these legacy POPs tend to be highly lipophilic and resistant to biodegradation [6]. The amphiphilic POPs such as PFASs are anthropogenic compounds, widely used in different commercial products since the 1940s, and are emerging POPs in the environment [7]. Therefore, both the legacy POPs and emerging PFASs bioaccumulate in the biota and biomagnifies in the food web. The arctic populations, including the Greenlandic Inuit, with traditional food intake including items from the marine food chain, such as seal, whale, walrus and polar bear [8,9], have a relatively high POP exposure. Metals, such as Hg, are not lipophilic, but the organic form, methyl-Hg, bioaccumulates in tissues [10,11,12]. The Arctic Monitoring and Assessment Programme (AMAP), established in 1991, aims to monitor POPs and heavy metals in the environment, animals, and humans in the Arctic [13].

Over the last decades, the concentrations of legacy POPs in humans have been decreasing due to the regulation of these chemicals, leading to reduced POPs found in the environment and marine food sources [14]. Human exposure to most legacy POPs is also decreasing in many Arctic populations, reflecting both transition from traditional to more imported westernized diet and reduced contaminant concentrations in the marine mammals [15]. However, within Greenland, there are strong regional differences, and Inuit who primarily rely on traditional marine mammals have relatively high contaminant concentrations [16]. Today, the Greenlandic Inuit population generally relies less on these traditional marine food sources and more on westernized imported foods and, accordingly, is relatively less exposed to the marine POP sources [16,17,18,19]. However, despite regulation of some POPs, it is still important to investigate and report the levels and adverse effects of the POPs, due to their long half-life and persisting high concentrations in the Arctic populations. Most of the emerging POPs, such as most PFASs and polybrominated diphenyl ethers (PBDEs), are not on the Stockholm Convention list but are used in industrial and commercial products. For the PFASs, only perfluorooctane sulfonate (PFOS) and perfluorooctanoic acid (PFOA) are on the Stockholm Convention list, and perfluorohexane sulfonate (PFHxS) has been reviewed and recommended for listing. For PBDE congeners, hexabrombiphenyl, heptabromodiphenyl ether, decabromodiphenyl ether (deca-BDE), hexabromocyclodecane (HBCDD), tetrabromodiphenyl ether and pentabromodiphenyl ether are on the Stockholm Convention list [5]. This makes studies of POPs highly relevant for future regulation.

Studies have shown that exposure to POPs, e.g., PCBs, OCPs, PFASs, and heavy metals, may be linked to immune system defects [20,21,22,23]; neurological effects, such as reduced IQ and behavioral abnormalities [24,25,26]; cancer [27]; birth defects; decreased fertility; altered sex hormone balance and altered metabolism [24,28,29,30,31]. POPs have hormone disrupting potentials and can act as agonist/antagonist on the estrogen-, androgen-, and aryl hydrocarbon receptors [32,33,34,35,36,37,38,39,40,41,42,43,44]. This is of particular concern for the developing organism being highly sensitive to hormonal changes and, thus, POP exposure, which can result in permanent changes in adult life [45]. Several studies have indicated that POPs can disrupt the endocrine system and can decrease the body’s defense against oxidative stress [24,46] and, therefore, are of concern regarding the public health status in Greenland.

Concentrations of various legacy POPs and heavy metals have been determined in blood from approximately 600 men and women from eight different Greenlandic districts during 1997 to 2006 [15]. The legacy POP concentrations in Inuit correlated with age, indicating age-related bioaccumulation of the compounds. Furthermore, men had higher serum concentrations of POPs and Hg, probably resulting from the ability of women to pass contaminants to their fetus and child through pregnancy and breastfeeding, respectively [24]. In addition, dietary preferences and amount consumed may play roles in this gender difference. Finally, as an indicator for marine mammal intake, the ratio of n−3 polyunsaturated fatty acids to n−6 polyunsaturated fatty acids (n−3/n−6) in the plasma was a predictor for the human legacy POP body burden [47,48,49,50,51,52].

During 1994 to 2006, longitudinal biomonitoring data of POPs and heavy metals from several Greenlandic districts was collected [17]. In 2010, being a part of the AMAP studies, the Adapting to Climate Change, Environmental Pollution and Dietary Transition (ACCEPT) birth cohort was established as a prospective and geographical mother–child cohort in Greenland [16], aiming at the exposure levels of contaminants, lifestyle and diet during pregnancy, and to assess possible interference with the fetal development and infant/child health. Between 2010 and 2015, the ACCEPT birth cohort enrolled 614 Greenlandic women and their children from different regions/districts in Greenland, and the concentrations of environmental contaminants, including legacy POPs, PFASs, PBDEs and metals in the blood of pregnant women, were measured [16,18,19].

The aim of the present study is to summarize the previous and recent biomonitoring investigations and determine the temporal trends of environmental contaminants in the blood of the Greenlandic population during 1994–2015.

## 2. Materials and Methods

### 2.1. Study Population

The subjects and sampling methods were described in detail elsewhere [16,18,19,49,50,53,54,55,56]. All participants were of Inuit decent, defined as having parents and/or grandparents born in Greenland. All participants completed a standard questionnaire, including questions about demographic and lifestyle parameters. Venous blood samples were prepared for determination of POPs, fatty acids and metals and stored at −80 °C until analyses as described [16,18,19,49,50,53,54,55,56].

**Figure 1:**
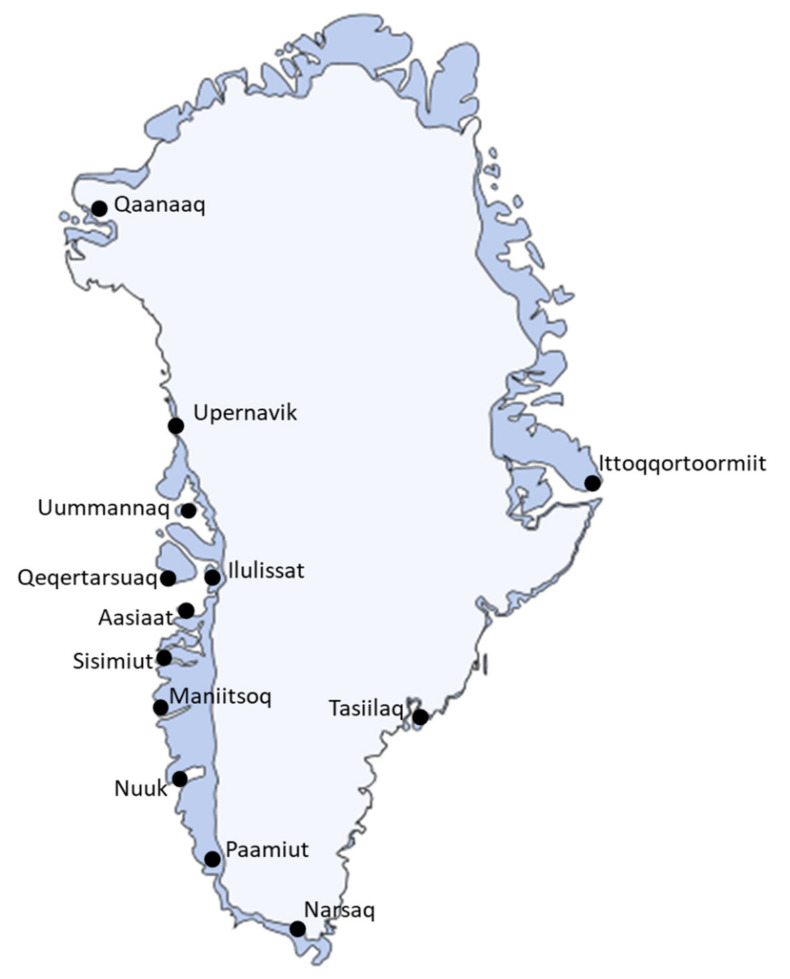
Map of Greenland with the collection sites.

In this study, the following districts were included: Qaanaaq, Upernavik, Uummannaq, Qeqertarsuaq, Ilulissat, Aasiaat, Sisimiut, Maniitsoq, Nuuk, Paamiut, Narsaq, Tasiilaq and Ittoqqortoormiit (Figure 1, Table 1).

### 2.2. Determination of POPs, Hg, Se and Fatty Acids

At the certificated laboratory Le Centre de Toxicologie du Québec, Quebec, Canada, serum samples were analyzed for legacy POPs by gas chromatography using a HP−5890 series II equipped with a dual column split–splitless injector and two electron capture detectors at 275 °C for the injector and 350 °C for the detectors. The detailed gas chromatography method has been described in [57]. The legacy POPs include cis-, trans- and oxy-chlordane; p,p’-DDE; p,p’-DDT; hexachlorobenzene; beta-Hexachlorocyclohexane; Mirex; toxaphene 26; toxaphene 50 and 14 PCB congeners (PCB28, PCB52, PCB99, PCB101, PCB105, PCB118, PCB128, PCB138, PCB153, PCB156, PCB170, PCB180, PCB183, PCB187), and the concentrations were given as µg/kg lipid upon adjustment to the serum lipid content [49,56,57].

Whole blood levels of total mercury (Hg), lead (Pb) and selenium (Se) were measured using Inductively Coupled Plasma Mass Spectrometry (ICP–MS) at the accredited element laboratory, the Institute for Bioscience—Arctic Research Centre, Aarhus University, Denmark [49,53], and Le Centre de Toxicologie du Québec, Québec, Canada [58,59]. The whole blood samples were dissolved and decomposed by nitric acid in Teflon bombs, and blood metals were measured by ICP–MS in an Agilent 7500 cs apparatus [48]. The precision and accuracy were checked by analyzing certified reference materials and by participation in the Quality Assurance of Information in Marine Environmental monitoring [60].

Serum levels of 16 PFASs were determined at the Department of Environmental Science, Aarhus University in Denmark. Previous reports give the details on analytical methods and specific compounds [16,56,61]. The PFASs include six perfluorinated sulfonic acids (PFSAs) (perfluorobutanesulfonic acid (PFBS, C4), perfluorohexane sulfonate (PFHxS, C6), perfluoroheptanesulfonate (PFHpS, C7), perfluorooctane sulfonate (PFOS, C8), perfluorooctane sulfonamide (PFOSA, C8), perfluorodecanesulfonate (PFDS, C10)) and ten perfluorinated carboxylic acids (PFCAs) (perfluoropentanoic acid (PFPeA, C5), perfluorohexanoic acid (PFHxA, C6), perfluoroheptanoic acid (PFHpA, C7), perfluorooctanoic acid (PFOA, C8), perfluorononanoic acid (PFNA, C9), perfluorodecanoic acid (PFDA, C10), perfluoroundecanoic acid (PFUnA, C11), perfluorododecanoic acid (PFDoA, C12), perfluorotridecanoic acid (PFTrA, C13), perfluorotetradecanoic acid (PFTeA, C14)).

The fatty acid profiles were determined in serum phospholipids at the Biology Department, University of Guelph, Canada [51]. The sum percentage of the n−3 polyunsaturated fatty acids are as follows: C18:3n3, C18:4n3, C20:3n3, C20:4n3, C20:5n3 eicosapentaenoic acid (EPA), C22:5n3 and C22:6n3 docosahexaenoic acid (DHA). The n−6 fatty acids are the sum percentage of C18:2n6, C18:3n6, C20:2n6, C20:3n6, C20:4n6 arachidonic acid (AA), C22:2n6, C22:4n6 and C22:5n6. The ratio between n−3 and n−6 is a strong indicator of marine food intake and gives the relative consumption of traditional marine food versus imported food [50,62].

### 2.3. Statistical Analysis: Temporal Trends Analyses

Temporal trend analyses were performed for the most commonly detected environmental contaminants, including the following compounds: PCB153, p,p’-DDE, oxychlordane, Hg, Pb, Se, PFHxS, PFOS, PFOA, PFNA, PFDA and PFUnA. The data is from the period 1994–2015.

Only time series with data from at least five years were included in the analyses of temporal trends. As shown in Table S1, in the present study, it was possible to make time trend analysis for the legacy lipophilic POP (termed as lipPOP: PCB 153, p,p’-DDE and oxychlordane) and metals (Hg, Pb, Se) for the women from the district Nuuk and Ilulissat. For the PFASs, only Nuuk women had data from at least five years. Moreover, we performed the time trend analysis for all women across thirteen Greenlandic districts of lipPOP, Hg, Pb, Se and PFASs. Fewer male participants were available for time series analysis in each district, and, therefore, we show data of the time trend analysis of lipPOP, metals and PFASs for all men across the Greenlandic districts. The time trend analyses of lipPOP, metals and PFASs are also given for the combined gender across Greenlandic districts.

The time trend analyses were performed by simple linear regression (i.e., the dependent variables were not ln-transformed) and ln-linear regression analysis (i.e., the dependent variables were ln-transformed). The assumption of linear regression regarding normality, linearity, homoscedasticity and absence of multicollinearity was checked by normal predicted probability (P-P) plot, scatterplot of the residuals and variance inflation factor (VIF) values. We tested the autocorrelation using the Durbin–Watson test. The ln-linear regression model fits the linear regression assumption better than the simple linear regression model; thus, we report the results of ln-linear regression analysis with ln-transformed dependent variables.

Preliminary analyses showed that age was highly related to contaminant concentrations, and the annual median age differs considerably among the study years. Thus, the temporal trend analyses were adjusted for age. The different lifestyles among districts may influence the levels of contaminants [18]; we therefore adjusted the temporal trend analyses of pooled women and pooled men across the Greenland districts for age and districts. The temporal trend analysis for the combined gender across the Greenlandic districts was adjusted for age, district and gender.

Among women, 51.1% were pregnant women (Table 2). Using analysis of covariance (ANCOVA), we compared the blood concentrations of compounds between pregnant women and nonpregnant women in the same sampling year by adjustment of age and districts. Considering physiologic changes during gestation, such as the body’s increasing blood volume, the serum lipid concentrations and concentrations of contaminants may decrease during the pregnancy [63], the temporal trend analysis of women data was further adjusted for pregnancy status. Body mass index (BMI) and n−3/n−6 ratio may also influence the levels of POPs and metals. However, more than 30% participants were missing such information (Table 2). Thus, BMI and n−3/n−6 were not included in the adjustments of analysis.

For sensitive analysis, we stratified the linear regression analysis by the median age. The following stratification were used: Nuuk women at 30 years old, Ilulissat women at 26 years old, women across Greenland at 29 years old and men across Greenland at 37 years old. In addition, we separately carried out the linear regression analysis for pregnant women and nonpregnant women.

Using the time trends of ln-transformed data, we estimate the time to halve (decline half times, T½) or to double (T2) the concentrations of serum of legacy POPs or PFASs, or blood metals. The T½ or T2 value was calculated by Ln(2)/β, where β was the slope obtained from the trend analysis [64,65].

The statistical analyses were performed in SPSS software 26.0 (SPSS Inc., Chicago, IL, USA) with a significance level of *p* < 0.05 and *p* < 0.10 as borderline significance.

## 3. Results

### 3.1. Study Population Characteristics

Figure 1 and Table 1 show the geographic distribution of the study population. The participants from Nuuk and Ilulissat accounted for the majority of the study population (37% and 23%), and 79.6% participants were women.

As shown in Table 2, most women in Nuuk were pregnant, while in Ilulissat, most women were nonpregnant. For the pooled data across all Greenlandic districts, the proportion of pregnant women and nonpregnant was similar (51% vs. 49%). The median age of Nuuk women was 30 years, while Ilulissat women had median age of 26 years. For the pooled data across all Greenlandic districts, male participants were older, compared to female participants, with a median of 37 vs. 29 years of age, respectively. The median BMI of the female participants in Nuuk and Ilulissat were normal, while male participants across the Greenlandic districts had slightly higher BMI than female participants, although 37% participants had no BMI information. Compared to male participants, the female participants had lower n−3/n−6 fatty acid ratio, which is a marine food intake biomarker, indicating lower intake of marine diet or higher intake of terrestrial food. However, 35% participants had no fatty acids data available.

### 3.2. Temporal Time Trend of Legacy Lipophilic POPs, Hg, Pb, Se and PFASs in Nuuk Women and Ilulissat Women

**Figure 2:**
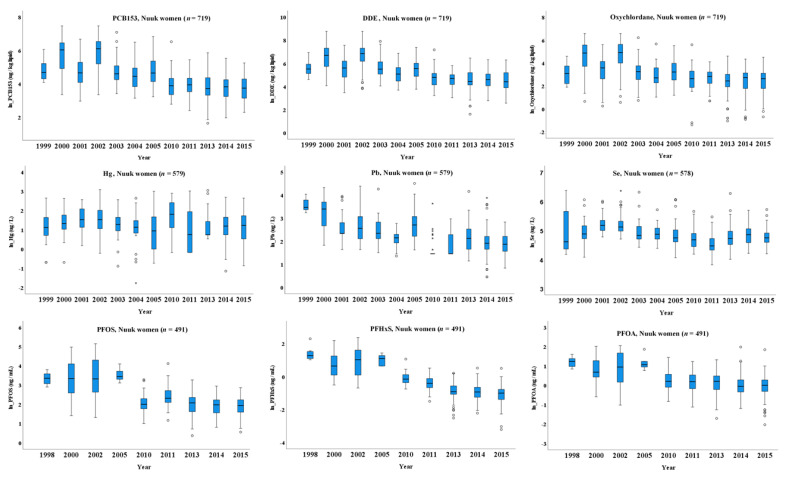

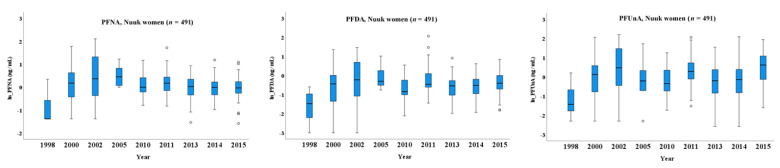
The time trend of lipPOPs, metals and PFASs in Nuuk women during 1998–2015. The boxes display the 25th and 75th centiles, and the line inside the boxes represents the median value. The whiskers display the lower and upper values within 1.5 times the interquartile range beyond the box. Outliers are marked with dots. The data were raw data and in ln-transformed scale. The figure in normal scale is given as Figure S1. Please see Table 2 for the adjusted data. LipPOP: legacy lipophilic POPs; PFASs: perfluoroalkylated substances. For the full name of chemicals, please refer to abbreviation list.

As shown in Table 3 and Figure 2 and Figure S1, Nuuk women had a significant declining trend for the legacy lipophilic POPs (lipPOP: PCB153, p,p’-DDE and oxychlordane), Hg, Pb and Se during 1999–2015 and PFHxS, PFOS and PFOA during 1998–2015. In the period of 1999–2015, after adjustment of age, the ln-transformed serum level of PCB153, p,p’-DDE, and oxychlordane decreased significantly by 0.09 µg/kg lipid per year, while ln-transformed blood level of Pb and Se significantly decreased 0.07 and 0.02 µg/L per year, respectively. Although age-adjusted blood Hg level showed nonsignificant decrease with year, after further adjustment of pregnancy status, a significant decreased trend of 0.02 µg/L per year was observed (Table 3). For Nuuk women during 1998–2015, upon age adjustment, the ln-transformed serum level of PFHxS, PFOS and PFOA decreased 0.12, 0.08 and 0.07 ng/ml per year, respectively. Serum PFNA, PFDA and PFUnA showed no significant trends, but the results suggest a borderline increasing tendency for PFDA and PFUnA. Further adjustment for pregnancy status elicited similar patterns (Table 3).

**Figure 3:**
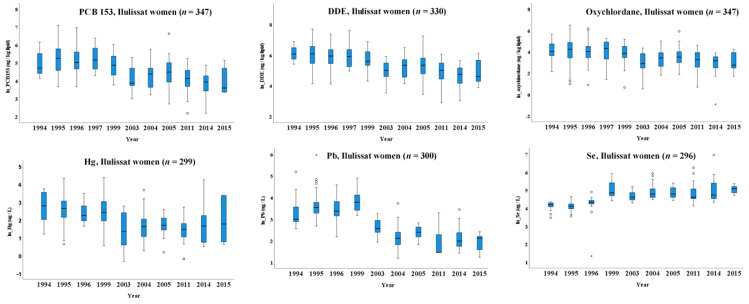
Time trend of lipPOPs and metals in Ilulissat women during 1994–2015. The boxes display the 25th and 75th centiles, and the line inside the boxes represents the median value. The whiskers display the lower and upper values within 1.5 times the interquartile range beyond the box. Outliers are marked with dots. The data are raw data and in ln-transformed scale. The figure in normal scale is given as Figure S2. Please see Table 2 for the adjusted data. LipPOP: legacy lipophilic POPs; for single POP congeners and metals, see abbreviation list.

In Ilulissat women, significant declining trends of PCB153, p,p’-DDE, oxychlordane, Hg and Pb were observed (Table 3, Figure 3 and Figure S2). During 1994–2015, upon age adjustment, the ln-transformed serum level of PCB153, p,p’-DDE, and oxychlordane decreased 0.07, 0.07 and 0.06 µg/kg lipid per year, respectively. Further adjustment for pregnancy showed a similar pattern. The age-adjusted ln-transformed blood level of Hg decreased 0.06 µg/L, and Pb decreased 0.10 µg/L per year (Table 3), whereas the age-adjusted ln-transformed blood level of Se increased 0.05 µg/L per year (Table 3, Figure 3). Further adjustment for pregnancy status showed similar patterns except for Hg, where the significant association disappeared (Table 3).

**Figure 4:**
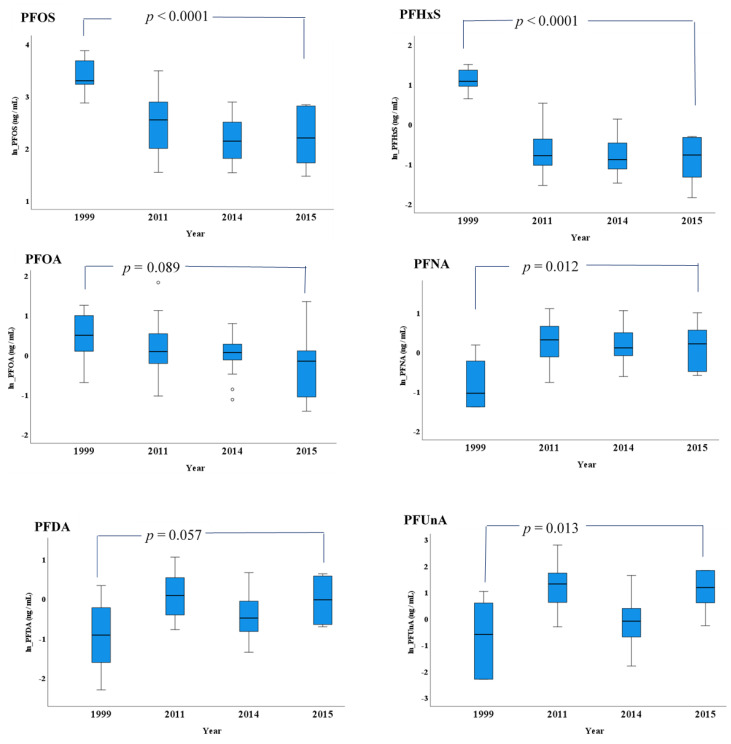
The levels of PFASs in Ilulissat women in 1999 (*n* = 10), 2011 (*n* = 33), 2014 (*n* = 20) and 2015 (*n* = 6). The boxes display the 25th and 75th centiles, and the line inside the boxes represents the median value. The whiskers display the lower and upper values within 1.5 times the interquartile range beyond the box. The data are raw data and in ln-transformed scale. The figure in normal scale is given as Figure S3. The p values are from analysis of covariance (ANCOVA) on ln-transformed data under the adjustment of age. PFASs: perfluoroalkylated substances. For the full name of single PFAS congeners, please refer to the abbreviation list.

Even though only data of four time points were available, we observed a declining trend for perfluorooctane sulfonate (PFOS), perfluorohexane sulfonate (PFHxS) and perfluorooctanoic acid (PFOA) in Ilulissat women during 1999–2015 (Figure 4 and Figure S3). The levels of PFOS and PFHxS in 2015 were significantly lower than that in 1999, while PFOA in 2015 was borderline lower than that in 1999. However, compared to 1999, the levels of perfluorononanoic acid (PFNA) and perfluoroundecanoic acid (PFUnA) were significantly higher, and PFDA borderline higher, in 2015 (Figure 4).

The time trend analysis, stratified by median age and adjusted for pregnancy status, showed a significantly declining trend of lipPOPs and metals for the Nuuk women, with the women over 30 years of age being slightly steeper for lipPOPs, compared to younger women, whereas the Hg decline was not significant in young Nuuk women (Table 4). In contrast, the significant declining trend of PFHxS was slightly steeper in younger Nuuk women **≤** 30 years of age, compared to women > 30 years of age. PFOS and PFOA were only significantly declining in the younger women (Table 4). For the Ilulissat women, the time trend of lipPOPs and metals were similar between women over 26 years of age and younger women **≤** 26 years of age, although there was no significant decrease for oxychlordane in women >26 years of age and Pb for younger women ≤26 years old (Table 4).

### 3.3. Time Trend of Legacy Lipophilic POPs, Hg, Pb, Se and PFASs in Women and Men across the Greenlandic Districts

Table 5 shows the temporal time trend of lipPOPs, metals and PFASs in all women and men across the Greenlandic districts. For women, we observed a significant decreasing trend of lipPOPs, Hg and Pb and slightly increasing trend of Se during 1994–2015. In the period 1997–2015, upon adjustment of age and district, ln-transformed concentration of PFHxS, PFOS and PFOA in women significantly decreased, with 0.11, 0.06 and 0.08 ng/mL per year, respectively, while ln-transformed concentration of PFNA, PFDA and PFUnA increased significantly, with 0.03, 0.05 and 0.05 ng/mL per year, although, for PFDA and PFUnA, the significance disappeared upon further adjustment for pregnancy (Table 5).

For Greenlandic men, Table 5 shows a significant declining trend of lipPOPs and metals during 1997–2006. After adjustment of age and districts, the each year decreased ln-transformed concentrations were 0.12 µg/kg lipid PCB153, 0.09 µg/kg lipid p,p’-DDE, 0.17 µg/kg lipid oxychlordane, 0.14 µg/L Hg, 0.13 µg/L Pb and 0.05 µg/L Se. For the PFASs, the yearly decreased ln-transformed concentrations were 0.10 ng/mL for PFHxS and PFOS, while PFOA and PFNA showed a borderline decrease trend (0.06 ng/mL per year). However, we found no significant changes over years for PFDA and PFUnA in the Greenlandic men (Table 5).

Table 6 shows, after stratifying by median age, the Greenlandic women older than 29 years elicited a steeper significant declining trend for lipPOPs, Hg, Pb, PFOS and PFOA than that of younger women, where the Hg decline was not significant upon further adjustment for pregnancy. We observed an increasing trend of PFNA, PFDA and PFUnA, significantly for younger women < = 29 years of age and being steeper than that of older women >29 years of age. Further adjustment for pregnancy status the association elicited similar data (Table 6).

As shown in Table S2, the declining trends were significant for both men who were younger and older than 37 years for lipPOPs, Hg and Pb after adjustment of district, while Se not being significant for men <= 37 years of age. However, the declining trends of lipPOPs and Pb was slightly steeper in men younger than 37 years old (Table S2).

When combining the men and women data across the Greenlandic districts, upon adjustment of age, districts and gender, we found significantly decreasing trends for lipPOPs, Hg, Pb and PFHxS, PFOS and PFOA but significant increasing trends for Se, PFNA, PFDA and PFUnA (Table S3).

### 3.4. The Annual Percentage Changes of Legacy Lipophilic POPs, Hg, Pb, Se and PFASs

Table 7 and Table 8 present the percentage changes in the concentration of POPs and metals in the serum/blood of the Greenlandic study population and the estimated elimination half-time or increase doubling time.

For Nuuk women, the most pronounced declines were observed for the lipPOPs (PCB153, p,p’-DDE, and oxychlordane), with an age-adjusted serum concentration decreased of 8.52%, 8.61% and 8.61% per year, respectively (Table 7). The estimated age-adjusted elimination half-times of PCB153, p,p’-DDE and oxychlordane were 7.79, 7.70 and 7.70 years, respectively. Hg level declined slowly in Nuuk women during 1999–2015, being significant upon adjustment for age and pregnancy. The age-adjusted concentration of Pb and Se decreased 6.48% and 2.18% per year with a calculated decrease half-time of 10.4 and 31.5 years, respectively. In Nuuk women, the age-adjusted concentration of PFHxS, PFOS and PFOA decreased 11.7%, 7.41% and 6.67% annually, with the decrease half-time of 5.59, 9.00 and 10.1 years, respectively (Table 7). The age-adjusted PFNA, PFDA and PFUnA levels showed a positive increase during 1998–2015. Although the positive trend was not significant upon further adjustment for pregnancy, the positive doubling time suggested increasing tendencies being borderline significance for PFDA and PFUnA (Table 7).

For the Ilulissat women during 1994–2015, the age-adjusted serum level of PCB153, p,p’-DDE, and oxychlordane decreased 6.85%, 6.67% and 6.11% per year, with the decline half-time of 9.76, 10.1 and 11.0 years, respectively (Table 7). Blood levels of Pb decreased more than Hg, and the decreased half-time was 7.00 years for Pb and 11.4 years for Hg. In contrast, blood Se level increased with 4.71% per year. Upon further adjustment of pregnancy, the significant decrease trend of Hg disappeared (Table 7).

As shown in Table 8, for all female participants across the Greenlandic districts, upon adjustment of age and district, the estimated elimination half-times were 7.7 years for PCB153 and p, p’-DDE, 6.93 years for oxychlordane, 9.9 years for Hg and 7.7 years for Pb, being slightly shorter compared to women in Nuuk and Ilulissat (Table 7). The calculated elimination half-times were 6.3 years for PFHxS, 11.6 years for PFOS and 8.66 years for PFOA (Table 8), being slightly longer compared to Nuuk women (Table 7). Table 8 shows the estimated doubling times were 23.1 years for PFNA and 13.9 years for PFDA and PFUnA, being shorter compared to Nuuk women (Table 7). The Se level of all women across Greenland weakly increased, with doubling time of 69.3 years (Table 8).

For male participants across Greenland during 1997–2006, the age and district-adjusted concentration of lipPOPs decreased 8.61–15.6% each year, and the decrease half-times were between 4.10–7.70 years. The heavy metals Hg and Pb decreased 13.1% and 12.2% each year during 1999–2006, and the calculated half-times were 5.00 and 5.30 years, respectively. The annual decrease of PFHxS, PFOS, PFOA and PFNA in men across Greenland were 9.52%, 9.52%, 5.82% and 5.82%, with the estimated half-time of 6.90, 6.90, 11.6 and 11.6 years, respectively (Table 8). Although nonsignificant, PFDA in men across Greenland showed a weakly increasing tendency during 1997–2006 (Table 8).

### 3.5. Sensitive Analysis

Among the female participants, 51% were pregnant. Since the physiological changes during the pregnancy may influence the measured concentration of the compounds, we compared the levels of POPs and metals of pregnant and nonpregnant women at the same sampling year by adjustment of age and districts. Overall, pregnant women showed lower levels than nonpregnant women (Table S4).

Considering the possible bias from pregnancy status, we further performed sensitive analyses for pregnant women and nonpregnant women separately (Table S5). For pregnant women, the results showed decreasing trends for PCB153, p,p’-DDE, oxychlordane, Hg, Pb and Se during 1999–2015 and PFHxS, PFOS, PFOA and PFNA during 2010–2015. However, for the nonpregnant women, PCB153, p,p’-DDE, oxychlordane, Hg and Pb elicited a decrease trend, while Se increased during 1994–2006. PFHxS, PFOS and PFOA showed a decrease trend in the period of 1997–2006. For neither pregnant nor nonpregnant women, we found no significant decline in time trends for PFDA and PFUnA (Table S5).

## 4. Discussion

POPs and heavy metals have persistent properties and can elicit harmful effects on environment and human health. The Stockholm Convention on POPs have currently listed 35 POPs, among which 26 POPs are listed in Annex A (Elimination), 2 in Annex B (Restriction) and 7 in Annex C (Unintentional production) [5]. Although the use of POPs has been either phased out or restricted, POPs are still present in the environment and in humans. The POP levels vary according to the geographic areas and populations.

We used time trend analyses to assess the stability of contaminants in the environment and the human blood over time. Moreover, trend data is also valuable to examine the impact of regulations and regulator policy regarding the elimination or restriction of adverse effects of environmental contaminants. Hence, detailed trend data are valuable.

In the present study, we report the time trend in the Greenlandic population of environment contaminants including legacy lipophilic POPs, Hg, Pb, Se and PFASs with the quantitative changes and percent changes per year.

### 4.1. Trend of Legacy Lipophilic POPs

We observed that the PCB 153 concentration decreased 8.52% per year for women in Nuuk during 1999–2015, 6.85% for women in Ilulissat and 8.61% for women across Greenlandic districts during 1994–2015. We found a steeper decrease in men across Greenland during 1997–2006, being 11.3% per year for PCB 153. The PCB153 decline in the present study was steeper than in Northern Norwegian women (3.9%) and men (4.0%) during 1986–2007 [66] but was comparable with Danish pregnant women (8.5% per year) during 2011–2013 [67]. Nevertheless, longer follow-up period and higher concentration of POPs in Greenlanders may partly explain this result. For example, in 2001, the median serum level of PCB153 of Nuuk women was 105 µg/kg lipid, while the level in Northern Norwegian women was 43 µg/kg lipid [66]. For the Danish women, the PCB153 level in 2011, 2012 and 2013 were 22 µg/kg lipid, 18 µg/kg lipid and 18 µg/kg lipid, respectively [67], being only the half concentration of the Greenlandic women, whose concentration were 57 µg/kg lipid in 2011 and 40 µg/kg lipid in 2013. Regulation must be a factor for both the Danish and Inuit women, but also the diet transition for the Greenlandic women might play a role in the lipPOP decrease over years.

The annual decrease in concentration of p,p’-DDE for women in Nuuk during 1999–2015 was 8.61%, similar with the annual decrease rate of 8.4% in Danish pregnant women in 2011–2013 [67]. Similar to previous reports of Arctic populations [14], we found a significant declining trend of oxychlordane in Greenlandic women and men, indicating the effectiveness of international conventions, as the oxychlordane precursor chlordane is under regulation and is included in the Stockholm Convention annex A [5]. The observed significant declining trends of the legacy lipophilic POPs, including PCB153, organochlorine pesticides, p,p’-DDE and oxychlordane, in Greenlandic females during 1994–2015 and males 1997–2006 is comparable with the previous reports from Arctic populations e.g., Nunavik of Canada, Yup’ik of Alaska, Iceland (Reykjavik), costal Chukotka of Russia, Disko Bay of Greenland [14] and Northern Norway [66]. Gibson et al. reported that most legacy lipophilic POPs have declined in the Arctic populations since 1986 [68]. The significant declining trends of legacy lipophilic POPs in the Arctic populations are the results of both the regulation to reduce production and usage of legacy lipophilic POPs since 1970 [69,70] and a reduction in consumption of traditional food in the Arctic being related to the increasing availability of commercial food [71]. The tendency to a steeper decline trend of PCB153, p’p-DDE and oxychlordane observed in the older women from Nuuk and all women across Greenland may be due to the higher amounts of POPs by age, because of their bioaccumulation properties. In addition, parity and breastfeeding may play role in this observation [72]. However, due to missing parity and breastfeeding information for some female participants, the adjustment of these two factors is impossible in the present study. For the Ilulissat women, the extent of the declining trend between younger and older was similar, and the possible explanation might be the more narrow age range, compared to Nuuk and all Greenland women (15–43 years vs. 15–66 years and 15–70 years).

### 4.2. Trend of Metals

Previous Arctic studies reported a declining trend of blood level of Hg in women from Nunavik of Canada (1992–2013), Faroe Islands (1998–2007), Disko Bay of Greenland (1994–2011) and Yup’ik of Alaska (2004–2012) [14,16,46,68]. However, Hg level in women from Nuuk in Greenland (1994–2011), Vasterbotten in Sweden (2004–2009) and Costal Chukokta in Russia (2001–2007) showed no significant changes [14,46]. In the present study, we observed that Hg level in Nuuk women showed a slightly but not significantly decreasing yearly trend from 1999–2015. After stratified by the median age, Nuuk women older than 30 years showed a slightly steeper decreasing trend, which may relate to their possible higher parity and breastfeeding duration than younger women [72]; however, due to missing information on parity and breastfeeding, the analysis was not adjusted for these parameters in the present study. The blood Hg level of the Ilulissat women significantly decreased over the period of 1994–2015, with the 5.92% decrease per year. When stratified by median age of 26 years, we found a decreasing trend for both younger and older women. Further adjustment of pregnancy changed the significance both for the Nuuk and Ilulissat women. However, it is not clear whether this change is due to the over-adjustment, as previous study demonstrated a lack of significant difference of Hg among the trimesters [73]. Further studies may elucidate the influence of pregnancy status on the body levels of contaminants.

We found a clear decreasing trend of blood Hg level for all the Greenlandic women during 1994–2015 and all the Greenlandic men across Greenland during 1999–2006 before and after adjustment of confounders. This may relate to the decreased intake of traditional food due to transition from traditional diet to a more westernize lifestyle and diet in Greenland. This is in accordance to the main source of Hg in the Arctic population of marine mammals [74], and the Hg concentrations in the Arctic marine mammal have decreased over the years [75,76]. Although declining, it should be kept in mind that Hg levels in Greenlanders are still higher than other Arctic populations, such as Alaska, Norway, Iceland, Finland and Russia [53,68,77].

The heavy metal Pb elicited a significantly decreasing trend in the Arctic populations between 1992 and 2013 [14,46]. We also observed Pb decreasing trends for Nuuk women during 1999–2015, Ilulissat women and all the women across Greenland during 1994–2015, as well as in the men across Greenland during 1997–2006. Fragments of lead shot in birds have been an important source of dietary Pb exposure. The decline of Pb in Greenlanders might relate to the banned lead shot in Greenland after 2012 and phase-out of leaded gasoline during 1990s [78,79]. Similar to legacy lipophilic POPs and Hg, the relatively steeper decline trend of Pb for older women compared to younger women may relate to the parity and breastfeeding.

The main source of the essential trace element Se for the Arctic population is also marine mammals [58]. Therefore, within the Arctic region, Se and heavy metals are generally analyzed together [74]. In the present study, we observed a declining trend of Se in Nuuk women and all men across Greenland; a possible explanation might be the decrease in intake of traditional marine food. Interestingly, we found for Ilulissat women and all women across Greenland significantly increasing trends of Se, with 4.71% and 1.01% per year during 1994–2015, respectively. However, Hg declined 5.92% in Ilulissat women and 6.76% in women across Greenland in the same period. Therefore, other sources than marine mammals might contribute to the body level Se in Greenlandic women, especially the Ilulissat women. A speculation could be that the regulation of Hg reduced the Hg release into the environment and, consequently, decreased the Hg level in the marine mammals and humans, while the essential element Se is stable in the environment and not affected by regulations. More studies are needed to elucidate the observed phenomena.

### 4.3. Trend of PFASs

Food intake is the major source of PFASs, but PFASs exposure through drinking water, food packaging, cooking procedure and household dust also contribute importantly to body levels [79,80]. Studies have shown that indoor environment accounted for up to 50% of PFASs intake of Norwegian women [81].

In the present study, we observed that regulated PFASs, PFOS and PFOA, as well as the proposed regulated PFHxS, elicited a significantly declining trend in both Nuuk women in 1998–2015 and all the women across Greenland during 1997–2015. The declining trends of PFOS and PFOA of Nuuk women older than 30 years and Greenland women older than 29 years were slightly steeper than those of younger women. This may relate to parity and breastfeeding, which can eliminate PFOS and PFOA from the body [82,83]. For the Ilulissat women, the levels PFOS, PFHxS and PFOA in 2015 were lower than that in 1999, suggesting a decrease tendency; however, too few data points were available to conduct time trend analyses. All the men across Greenland during 1997–2006 also showed a declining trend. Long et al. reported that PFOS elicited a slightly increasing trend in Nuuk women during 1998–2005, while we found, after adjustment for age, a declining nonsignificant trend [61]. This may suggest that PFOS levels in the Nuuk women started to decline since 2005, supported by the unadjusted data showed in Figure 2 and by Wielsøe et al. reporting that, for Greenlandic women, PFOS level in the period of 2011–2014 were significantly lower than in the period of 2000–2003 [84].

To avoid bias resulting from age and pregnancy, further adjustments for age and pregnancy showed a significant decreasing trend of PFOS, PFHxS and PFOA persisted in Nuuk women and all women across Greenland. The PFOS level decreased in Nunavik women during 2004–2012 [14,46]. Moreover, declining trends of PFOS and PFOA were reported for Swedish women from 1996–2009 [14,85]. The declining trends of PFOS and PFOA might be related to the international intervention, as PFOS was listed as a restriction POP and PFOA as an elimination POP in the Stockholm Convention [5] and phase-out action from the industry 3M started around 2000 [85,86]. However, in women from Yup’ik of Alaska, the concentrations of PFOS and PFOA were higher in 2009–2012 than in 2004–2006 [14,46]. Hence, despite the phase-out of listed POPs production and usage, contaminant recirculation in the environment through the environmental fate will remain. PFHxS is still under review by the POPs review committee [87]. A study reported an increasing trend of PFHxS during 1996–2010 in Swedish women [85], while a report on Danish pregnant women during 2008–2013 showed a declining trend [64]. Similar to Danish pregnant women, we also observed that PFHxS level in Greenlandic women in Nuuk, Ilulissat and across Greenland significantly decreased from 1998 to 2015. We found also a decreasing trend of PFHxS in all men across Greenland during 1997–2006. In support of our observation, European studies report a PFHxS decrease starting around 2000 [64]. The differences in temporal trends of PFHxS between studies may be due to differences in study design (e.g., the investigated period, study population) and regional differences in production, exposure and use of PFHxS-related compounds and/or products containing the compounds. PFHxS is a residual byproduct in the production of perfluorohexane sulfonyl fluoride (PHFxSF), which is a PFHxS precursor used in firefighting foams and postmarked carpet treatment applications. In addition to diet, human exposure to PFHxS can be from consumer products and dust [85].

In a previous report, PFNA elicited an increasing trend in women from Yup’ik of Alaska and Nunavik, Canada [14,46]. In the present study, we found, when pooling all women data across the Greenlandic districts, a significant increasing trend of PFNA both before and after confounder adjustment. We did not observe a significant increasing trend of PFNA for the Nuuk women during 1998–2015, while we found significantly higher PFNA levels in the Ilulissat women in 2015 compared to 1999. PFNA is not on the Stockholm Convention POP list, and that can explain the increasing trend. The increasing trend of PFNA might also be due to the continued production after 2001 [88], along with longer elimination half-lives and bioaccumulation ability in relation to the shorter chain PFASs [89,90], as PFNA (C9) has a longer carbon chain than PFOA and PFOS (C8).

In serum of Northern Norway adult men, PFNA, PFDA and PFUnA continuously increased during 1997–2007 [91]. In the Greenlandic adult male in the present study, it differs from the increasing PFNA trend observed among Northern Norway men, as PFNA level in men across Greenland decreased, while PFDA and PFUnA levels were stable from 1997 to 2006. This might relate to differences in the exposure profile and exposure level. As the present trend analysis only included 74 Greenlandic men, time series data including more men from Greenland might confirm this observation.

For the long chain PFASs, PFDA and PFUnA, we found an increasing trend for women either in Nuuk, Ilulissat or across Greenlandic districts during 1998–2015. It was reported an increasing trend of PFDA but no change for PFUnA in Swedish primiparous women between 1996 and 2010 [85]. The increase and/or stable trend of PFDA and PFUnA address the importance to take into account the regulation of these long chain PFASs.

## 5. Conclusions

The present study showed that, over the time, the levels of legacy lipophilic POPs, such as PCB153, p,p’-DDE and oxychlordane, have significantly declined in Greenlandic women and men, reflecting both transition from traditional to westernized diet and reduced concentrations in the marine mammals because of regulation. The PFASs listed on the Stockholm Convention list, PFOS and PFOA, and the proposed listing of PFHxS showed clear declining trends in women and men. This suggests the effectiveness of international and national regulation/notification and indicates the importance of global actions to reduce the release of contaminants. On the other hand, no obvious declining trend was observed for the serum level of the longer chain PFASs, such as PFNA, PFDA and PFUnA in the Greenlandic population, suggesting a concern about PFASs not being regulated currently, and should be taken into account.

Regarding the heavy metals, we observed a significant declining trend of Pb for women from Nuuk, Ilulissat, and all women and men across Greenland. Moreover, we found a declining trend of blood level of Hg for Ilulissat women and women across Greenland since 1994 and Greenlandic men during 1997–2006. However, the decline of Hg in Nuuk women was weak. The time trend of the trace element Se varies between geographical regions of Greenland and gender. We found that the Se level in Nuuk women significantly declined during 1998–2015 but showed an increasing trend for the Ilulissat women and women across Greenland. The different contributions from traditional food, such as marine mammals and other sources, may influence the time trend of different environment contaminants and essential trace element.

The time trend analyses in the Greenlandic study population indicate a decline in the concentrations of regulated POPs and heavy metals. In contrast, several nonregulated PFAS compounds (PFNA, PFDA and PFUnA) showed no significant declining trend, but a yearly increase in women across Greenland. Further studies with more recent exposure data must follow the trend for these compounds. The potential implications for human health highlight the need to continue biomonitoring of contaminants of concern. Further biomonitoring will also provide evidence and data for risk management strategies to protect the Arctic population heath.

## Figures and Tables

**Table 1:** The sample number of study population in Greenland during 1994–2015.

Town/District	LipPOP, Metal Dataset (1994–2015)		PFAS Dataset (1997–2015)	
Qaanaaq	82	(men: 43, women: 39)	33	(men: 13, women: 20)
Upernavik	11	(men: 11, women: 0)	2	(men: 2, women: 0)
Uummannaq	107	(men: 74, women: 33)	26	(men: 15, women: 11)
Qeqertarsuaq	80	(men: 35, women: 45)	20	(men: 10, women: 10)
Ilulissat	430	(men: 14, women: 416)	78	(men: 9, women: 69)
Aasiaat	31	(men: 0, women: 31)	31	(men: 0, women: 31)
Sisimiut	161	(men: 52, women: 109)	74	(men: 7, women: 67)
Maniitsoq	50	(men: 0, women: 50)	39	(men: 0, women: 39)
Nuuk	794	(men: 61, women: 733)	496	(men: 5, women: 491)
Paamiut	4	(men: 0, women: 4)	4	(men: 0, women: 4)
Narsaq	74	(men: 32, women: 42)	10	(men: 0, women: 10)
Tasiilaq	115	(men: 40, women: 75)	29	(men: 3, women: 26)
Ittoqqortoormiit	110	(men: 67, women: 43)	19	(men: 11, women: 8)

LipPOP: legacy lipophilic persistent organic pollutants (POPs) include polychlorinated biphenyls (PCBs) and organochlorine pesticides (OCPs) regulated by the Stockholm Convention list. PFASs: perfluoroalkylated substances.

**Table 2:** Characteristics of Greenlandic participants.

Parameter		District								
		Nuuk			Ilulissat			All Greenlandic Districts ^1^		
		Men	Women	All	Men	Women	All	Men	Women	All
Sampling year		1997–2005	1999–2015	1997–2015	1997	1994–2015	1994–2015	1997–2015	1994–2015	1994–2015
	*N*	61	733	794	14	478	492	434	1693	2131
	Pregnant Nonpregnant	- -	566 167	566 167	- -	116 362	116 362	- -	865 828	865 828
Age (year)	*n*	61	733	794	14	416	430	429	1620	2049
	Median	51	30	30	43	26	26	37	29	30
	Min-Max	20–77	15–66	15–77	23–59	15–43	15–59	18–79	15–70	15–79
	*Missing n (%)*	*0(0)*	*0(0)*	*0(0)*	*0(0)*	*62(13.0)*	*62(12.6)*	*5(1.2)*	*73(4.3)*	*82(3.8)*
BMI (kg/m^2^)	*n*	46	505	551	NA	54	54	363	973	1336
	Median	27.8	24.9	25	NA	23.3	23.3	26.4	25	25.3
	Min-Max	21.9–35.4	16.6–43.4	16.6–43.4	NA	17.7–39.4	17.7–39.4	17.6–41.0	16.4–47.2	16.4–47.2
	*Missing n (%)*	*15(24.6)*	*228(31.1)*	*243(30.6)*	*14(100)*	*424(88.7)*	*438(89.0)*	*71(16.4)*	*720(42.5)*	*795(37.3)*
n−3/n−6	*n*	50	524	574	NA	59	59	366	1012	1382
	Median	0.59	0.24	0.26	NA	0.23	0.23	0.39	0.26	0.28
	Min-Max	0.20–1.93	0.07–2.21	0.07–2.21	NA	0.11–0.51	0.11–0.51	0.09–1.93	0.07–2.21	0.07–2.21
	*Missing n (%)*	*11(18.0)*	*209(28.5)*	*220(27.7)*	*14(100)*	*419(87.7)*	*433(88.0)*	*68(15.7)*	*681(40.2)*	*749(35.1)*

^1^ The included Greenlandic districts were Qaanaaq, Upernavik, Uummannaq, Qeqertarsuaq, Ilulissat, Aasiaat, Sisimiut, Maniitsoq, Nuuk, Paamiut, Narsaq, Tasiilaq and Ittoqqortoormiit. BMI: body mass index; N: number of total participants; n: number of participants with information on the parameter; n−3: n−3 polyunsaturated fatty acids; n−6: n−6 fatty acids; NA: not available; Min: minimum; Max: maximum. The number and percentage of missing data are given in italics.

**Table 3:** Women in Nuuk and Ilulissat: Time trend of lipPOPs, metals and PFASs level.

Nuuk Women (1999–2015), Median Age 30 Years (min-max: 15–66)												
Chemical	Raw				Adjusted ^a^				Adjusted ^b^			
	*n*	β	95% CI	*p*	*n*	β	95% CI	*p*	*n*	β	95% CI	*p*
LipPOPs (µg/kg lipid)												
ln PCB153	719	−0.13	−0.15; −0.12	**<0.0001**	719	−0.09	−0.10; −0.08	**<0.0001**	719	−0.09	−0.10; −0.07	**<0.0001**
ln *p,p’*-DDE	719	−0.13	−0.15; −0.12	**<0.0001**	719	−0.09	−0.10; −0.08	**<0.0001**	719	−0.09	−0.10; −0.08	**<0.0001**
ln Oxychlordane	719	−0.14	−0.16; −0.12	**<0.0001**	719	−0.09	−0.11; −0.08	**<0.0001**	719	−0.09	−0.10; −0.07	**<0.0001**
Metals (µg/L)												
ln Hg	578	−0.01	−0.02; 0.003	0.129	578	−0.009	−0.02; 0.003	0.140	578	−0.02	−0.03; −0.003	**0.010**
ln Pb	578	−0.07	−0.08; −0.06	**<0.0001**	578	−0.07	−0.08; −0.06	**<0.0001**	578	−0.07	−0.07; −0.06	**<0.0001**
ln Se	578	−0.02	−0.03; −0.02	**<0.0001**	578	−0.02	−0.03; −0.02	**<0.0001**	578	−0.02	−0.03; −0.02	**<0.0001**
Nuuk women (1998–2015), Median age 30 years (min-max: 17–66)												
PFASs (ng/mL)												
ln PFHxS	491	−0.14	−0.15; −0.13	**<0.0001**	481	−0.12	−0.15; −0.10	**<0.0001**	481	−0.15	−0.18; −0.11	**<0.0001**
ln PFOS	491	−0.11	−0.12; −0.10	**<0.0001**	481	−0.08	−0.10; −0.05	**<0.0001**	481	−0.06	−0.10; −0.02	**0.002**
ln PFOA	491	−0.07	−0.08; −0.06	**<0.0001**	481	−0.07	−0.09; −0.05	**<0.0001**	481	−0.05	−0.08; −0.01	**0.010**
ln PFNA	491	−0.01	−0.02; 0.001	0.074#	481	0.002	−0.02; 0.03	0.845	481	−0.01	−0.05; 0.02	0.439
ln PFDA	491	0.001	−0.01; 0.01	0.863	481	0.02	−0.004; 0.05	0.090#	481	0.02	−0.02; 0.07	0.326
ln PFUnA	491	−0.001	−0.02; 0.02	0.885	481	0.04	−0.001; 0.07	0.058#	481	0.03	−0.03; 0.09	0.338
Ilulissat women (1994–2015), Median age 26 years (min-max: 15–43)												
LipPOPs (µg/kg lipid)												
ln PCB153	347	−0.07	−0.09; −0.06	**<0.0001**	347	−0.07	−0.08; −0.06	**<0.0001**	346	−0.05	−0.08; −0.03	**<0.0001**
ln *p,p’*-DDE	330	−0.07	−0.08; −0.06	**<0.0001**	329	−0.07	−0.08; −0.06	**<0.0001**	329	−0.07	−0.08; −0.06	**<0.0001**
ln Oxychlordane	347	−0.07	−0.08; −0.05	**<0.0001**	346	−0.06	−0.08; −0.05	**<0.0001**	346	−0.05	−0.09; −0.01	**0.020**
Metals (µg/L)												
ln Hg	299	−0.06	−0.08; −0.05	**<0.0001**	298	−0.06	−0.08; −0.05	**<0.0001**	298	−0.007	−0.04; 0.03	0.679
ln Pb	300	−0.10	−0.11; −0.09	**<0.0001**	299	−0.10	−0.11; −0.09	**<0.0001**	299	−0.02	−0.04; −0.003	**0.020**
ln Se	296	0.05	0.04; 0.05	**<0.0001**	295	0.05	0.04; 0.05	**<0.0001**	295	0.04	0.03; 0.06	**<0.0001**

LipPOP: legacy lipophilic POPs; PFASs: perfluoroalkylated substances; ^a^ adjusted for age. ^b^ adjusted for age and pregnancy status (Nuuk pregnant women *n* = 554 for lipPOPs and metals, *n* = 354 for PFASs; Ilulissat pregnant women: *n* = 111 for lipPOP and metals). Bold indicate statistical significance (*p* < 0.05). ^#^: Borderline significant (0.05 < *p* < 0.10). *n*: number of participants in the analyses. Because of only four time series of PFASs for Ilulissat women, the PFAS time trend analysis was not carried out (see Figure 4 for trend data). For the full name of chemicals, please refer to the abbreviation list.

**Table 4:** Nuuk and Ilulissat women: Age stratified time trend of lipPOPs, metals and PFASs level.

Chemical	Nuuk Women (1999–2015), Median Age 30 Years (Min-Max: 15–66)															
	≤ 30 Years								>30 Years							
	Raw				Adjusted ^a^				Raw				Adjusted ^a^			
	*n*	β	95% CI	*p*	*n*	β	95% CI	*p*	*n*	β	95% CI	*p*	*n*	β	95% CI	*p*
LipPOPs (µg/kg lipid)																
ln PCB153	387	−0.08	−0.09; −0.06	**<0.0001**	387	−0.08	−0.09; −0.06	**<0.0001**	333	−0.17	−0.18; −0.15	**<0.0001**	333	−0.11	−0.13; −0.09	**<0.0001**
ln *p,p’*-DDE	386	−0.08	−0.09; −0.06	**<0.0001**	386	−0.08	−0.09; −0.06	**<0.0001**	333	−0.16	−0.18; −0.15	**<0.0001**	333	−0.10	−0.12; −0.08	**<0.0001**
ln Oxychlordane	386	−0.07	−0.08; −0.05	**<0.0001**	386	−0.06	−0.08; −0.05	**<0.0001**	333	−0.20	−0.22; −0.17	**<0.0001**	333	−0.13	−0.16; −0.10	**<0.0001**
Metals (µg/L)																
ln Hg	377	−0.004	−0.02; 0.01	0.614	377	−0.01	−0.02; 0.01	0.464	201	−0.02	−0.04; 0.003	0.084#	201	−0.04	−0.06; −0.01	**0.002**
ln Pb	377	−0.06	−0.07; −0.05	**<0.0001**	377	−0.06	−0.07; −0.05	**<0.0001**	201	−0.08	−0.10; −0.06	**<0.0001**	201	−0.07	−0.09; −0.05	**<0.0001**
ln Se	377	−0.02	−0.03; −0.01	**<0.0001**	377	−0.02	−0.03; −0.01	**<0.0001**	201	−0.03	−0.04; −0.02	**<0.0001**	201	−0.03	−0.04; −0.02	**<0.0001**
Nuuk women (1998–2015), Median age 30 years (min-max: 17–66)																
PFASs (ng/mL)																
ln PFHxS	246	−0.19	−0.23; −0.15	**<0.0001**	246	−0.19	−0.23; −0.15	**<0.0001**	235	−0.13	−0.14; −0.11	**<0.0001**	235	−0.09	−0.15; −0.02	**0.010**
ln PFOS	246	−0.06	−0.10; −0.02	**0.003**	246	−0.06	−0.10; −0.02	**0.003**	235	−0.11	−0.12; −0.09	**<0.0001**	235	−0.06	−0.13; 0.01	0.105
ln PFOA	246	−0.05	−0.10; −0.01	**0.019**	246	−0.05	−0.10; −0.01	**0.019**	235	−0.07	−0.08; −0.06	**<0.0001**	235	−0.04	−0.10; 0.02	0.213
ln PFNA	246	−0.02	−0.06; 0.01	0.226	246	−0.02	−0.06; 0.01	0.226	235	−0.02	−0.03; 0.002	0.076 ^#^	235	−0.01	−0.08; 0.07	0.890
ln PFDA	246	0.02	−0.03; 0.06	0.505	246	0.02	−0.03; 0.06	0.505	235	−0.01	−0.02; 0.01	0.607	235	0.03	−0.05; 0.12	0.427
ln PFUnA	246	0.04	−0.03; 0.11	0.299	246	0.04	−0.03; 0.11	0.299	235	−0.01	−0.03; 0.01	0.326	235	0.01	−0.09; 0.11	0.827
Chemical	Ilulissat women (1994–2015), Median age 26 years (min-max: 15–43)															
	≤26 years								>26 years							
	Raw				Adjusted ^a^				Raw				Adjusted ^a^			
	*n*	β	95% CI	*p*	*n*	β	95% CI	*p*	*n*	β	95% CI	*p*	*n*	β	95% CI	*p*
LipPOPs (µg/kg lipid)																
In PCB153	184	−0.07	−0.08; −0.05	**<0.0001**	184	−0.05	−0.08; −0.01	**0.014**	162	−0.08	−0.09; −0.06	**<0.0001**	162	−0.06	−0.10; −0.01	**0.014**
ln *p,p’*-DDE	177	−0.07	−0.09; −0.05	**<0.0001**	177	−0.06	−0.10; −0.02	**0.004**	152	−0.07	−0.09; −0.05	**<0.0001**	152	−0.05	−0.09; 0.00	**0.049**
ln Oxychlordane	184	−0.06	−0.08; −0.04	**<0.0001**	184	−0.05	−0.10; −0.01	**0.031**	162	−0.07	−0.09; −0.04	**<0.0001**	162	−0.04	−0.10; 0.03	0.294
Metals (µg/L)																
ln Hg	158	−0.07	−0.09; −0.05	**<0.0001**	158	−0.02	−0.06; 0.02	0.319	140	−0.05	−0.07; −0.03	**<0.0001**	140	0.01	−0.04; 0.06	0.723
ln Pb	158	−0.10	−0.11; −0.09	**<0.0001**	158	−0.02	−0.04; 0.01	0.289	141	−0.10	−0.11; −0.09	**<0.0001**	141	−0.03	−0.07; −0.004	**0.027**
ln Se	156	0.04	0.03; 0.05	**<0.0001**	156	0.04	0.02; 0.07	**<0.0001**	139	0.05	0.04; 0.06	**<0.0001**	139	0.05	0.01; 0.08	**0.005**

LipPOP: legacy lipophilic POPs; PFASs: perfluoroalkylated substances; ^a^ Data was adjusted for pregnancy status (Nuuk pregnant women: age ≤ 30 years: *n* = 338 for lipPOPs and metals, *n* = 246 for PFASs; age > 30 years: *n* = 176 for lipPOPs and metals, *n* = 169 for PFAS data set. Ilulissat pregnant women: age ≤ 26 years: *n* = 64; age > 26 years: *n* = 46 for lipPOPs and metals). *n*: number of participants in the analysis. Bold indicate statistical significance (*p* <0.05). ^#^: Borderline significant (0.05 < *p* < 0.10). PFASs in Ilulissat participants were not performed time trend analysis stratified by age due to small sample size (*n* = 79) and only had four time series. For the full name of chemicals, please refer to the abbreviation list.

**Table 5:** **Women and Men across Greenland**: Time trend of lipPOPs, metals and PFASs levels.

Greenland Women (1994–2015), Median Age 29 Years (Min-Max: 15–70)												
Chemical	Raw				Adjusted ^a^				Adjusted ^b^			
	*n*	β	95% CI	*p*	*n*	β	95% CI	*p*	*n*	β	95% CI	*p*
LipPOPs (µg/kg lipid)												
ln PCB153	1522	−0.10	−0.11; −0.09	**<0.0001**	1512	−0.09	−0.10; −0.08	**<0.0001**	1512	−0.06	−0.07; −0.05	**<0.0001**
ln *p,p’*-DDE	1504	−0.10	−0.10; −0.09	**<0.0001**	1494	−0.09	−0.09; −0.08	**<0.0001**	1494	−0.06	−0.07; −0.05	**<0.0001**
ln Oxychlordane	1506	−0.11	−0.12; −0.10	**<0.0001**	1496	−0.10	−0.11; −0.09	**<0.0001**	1496	−0.06	−0.08; −0.05	**<0.0001**
Metals (µg/L)												
ln Hg	1315	−0.07	−0.08; −0.06	**<0.0001**	1304	−0.07	−0.08; −0.06	**<0.0001**	1304	−0.02	−0.03; −0.01	**<0.0001**
ln Pb	1311	−0.09	−0.10; −0.09	**<0.0001**	1300	−0.09	−0.10; −0.09	**<0.0001**	1300	−0.06	−0.07; −0.06	**<0.0001**
ln Se	1312	0.01	0.004; 0.01	**<0.0001**	1301	0.01	0.01; 0.02	**0.001**	1301	0.01	0.01; 0.02	**0.001**
Greenland Women (1997–2015), Median age 29 years (min-max: 16–66)												
PFASs (ng/mL)												
ln PFHxS	792	−0.12	−0.13; −0.11	**<0.0001**	763	−0.11	−0.13; −0.10	**<0.0001**	763	−0.16	−0.19; −0.13	**<0.0001**
ln PFOS	792	−0.09	−0.09; −0.08	**<0.0001**	763	−0.06	−0.07; −0.04	**<0.0001**	763	−0.09	−0.12; −0.06	**<0.0001**
ln PFOA	792	−0.04	−0.05; −0.03	**<0.0001**	763	−0.08	−0.11; −0.05	**<0.0001**	763	−0.08	−0.11; −0.05	**<0.0001**
ln PFNA	792	0.02	0.01; 0.03	**0.001**	763	0.03	0.02; 0.05	**<0.0001**	763	−0.05	−0.08; −0.02	**0.001**
ln PFDA	792	0.03	0.02; 0.04	**<0.0001**	763	0.05	0.04; 0.07	**<0.0001**	763	−0.02	−0.05; 0.02	0.364
ln PFUnA	792	0.03	0.02; 0.05	**<0.0001**	763	0.05	0.03; 0.07	**<0.0001**	763	−0.03	−0.07; 0.02	0.250
Greenland Men (1997–2006), Median age 37 years (min-max: 18–79)												
LipPOPs (µg/kg lipid)												
ln PCB153	400	−0.09	−0.12; −0.06	**<0.0001**	400	−0.12	−0.15; −0.09	**<0.0001**				
ln *p,p’*-DDE	400	−0.05	−0.07; −0.02	**0.001**	400	−0.09	−0.11; −0.06	**<0.0001**				
ln Oxychlordane	386	−0.11	−0.15; −0.07	**<0.0001**	386	−0.17	−0.20; −0.13	**<0.0001**				
Metals (µg/L)												
ln Hg	349	−0.04	−0.08; −0.01	**0.018**	349	−0.14	−0.17; −0.11	**<0.0001**				
ln Pb	346	−0.10	−0.12; −0.08	**<0.0001**	346	−0.13	−0.16; −0.11	**<0.0001**				
ln Se	350	0.02	−0.01; 0.04	0.289	350	−0.05	−0.08; −0.03	**<0.0001**				
PFASs (ng/mL)												
ln PFHxS	74	−0.12	−0.17; −0.07	**<0.0001**	73	−0.10	−0.17; −0.03	**0.004**				
ln PFOS	74	−0.14	−0.20; −0.08	**<0.0001**	73	−0.10	−0.16; −0.04	**0.002**				
ln PFOA	74	−0,13	−0,20; −0.07	**<0.0001**	73	−0.06	−0.14; 0.01	0.097 ^#^				
ln PFNA	74	−0.07	−0.13; −0.005	**0.040**	73	−0.06	−0.12; 0.01	0.081 ^#^				
ln PFDA	74	−0.05	−0.13; 0.04	0.272	73	0.01	−0.07; 0.09	0.872				
ln PFUnA	74	−0.05	−0.13; 0.04	0.275	73	−0.04	−0.13; 0.04	0.327				

LipPOP: legacy lipophilic POPs; PFASs: perfluoroalkylated substances; ^a^ adjusted for age and district; ^b^ adjusted for age, district and pregnancy status (pregnant women: *n* = 842 for lipPOPs and metals, *n* = 583 for PFASs). *n*: number of participants in the analysis. Bold indicate statistical significance (*p* <0.05), ^#^: borderline significance (0.05 < *p* <0.10). The Greenland women data were collected from the following districts: Qaanaaq (*n* = 37), Uummannaq (*n* = 16), Qeqertarsuaq (*n* = 45), Ilulissat (*n* = 408), Aasiaat (*n* = 29), Sisimiut (*n* = 109), Maniitsoq (*n* = 46), Nuuk (*n* = 720), Paamiut (*n* = 4), Narsaq (*n* = 42), Tasiilaq (*n* = 75) and Ittoqqortoormiit (*n* = 43). The Greenland men data were collected from the following districts: Qaanaaq (*n* = 41), Upernavik (*n* = 11), Uummannaq (*n* = 62), Qeqertarsuaq (*n* = 35), Ilulissat (*n* = 14), Sisimiut (*n* = 51), Nuuk (*n* = 49), Narsaq (*n* = 30), Tasiilaq (*n* = 40) and Ittoqqortoormiit (*n* = 67). For the full name of chemicals, please refer to the abbreviation list.

**Table 6:** **Women across Greenland**: age stratified time trend of lipPOPs, metals and PFASs levels.

Age Group	Greenland Women (1994–2015), Median Age 29 Years (min-max: 15–70)												
	Chemical	Raw				Adjusted ^a^				Adjusted ^b^			
		*n*	β	95% CI	*p*	*n*	β	95% CI	*p*	*n*	β	95% CI	*p*
≤29 years	LipPOPs (µg/kg lipid)												
	ln PCB153	803	−0.07	−0.08; −0.06	**<0.0001**	797	−0.07	−0.08; −0.07	**<0.0001**	797	−0.06	−0.07; −0.04	**<0.0001**
	ln *p,p’*-DDE	792	−0.07	−0.08; −0.06	**<0.0001**	786	−0.07	−0.08; −0.07	**<0.0001**	786	−0.06	−0.07; −0.04	**<0.0001**
	ln Oxychlordan	802	−0.07	−0.08; −0.06	**<0.0001**	796	−0.07	−0.08; −0.06	**<0.0001**	796	−0.05	−0.06; −0.03	**<0.0001**
	Metals (µg/L)												
	ln Hg	757	−0.06	−0.07; −0.05	**<0.0001**	750	−0.06	−0.07; −0.05	**<0.0001**	750	−0.01	−0.02; 0.01	0.445
	ln Pb	757	−0.09	−0.09; −0.08	**<0.0001**	750	−0.09	−0.10; −0.08	**<0.0001**	750	−0.06	−0.07; −0.05	**<0.0001**
	ln Se	756	0.02	0.01; 0.02	**<0.0001**	749	0.02	0.01; 0.03	**<0.0001**	749	0.02	0.01; 0.02	**<0.0001**
	PFASs (ng/mL)												
	ln PFHxS	391	−0.12	−0.14; −0.10	**<0.0001**	385	−0.12	−0.15; −0.10	**<0.0001**	385	−0.16	−0.20; −0.13	**<0.0001**
	ln PFOS	391	−0.02	−0.04; 0.001	0.060#	385	−0.03	−0.05; −0.01	**0.009**	385	−0.06	−0.10; −0.03	**0.010**
	ln PFOA	391	0.03	0.002; 0.05	**0.035**	385	**0.02**	−0.01; 0.04	0.131	385	−0.06	−0.09; −0.02	0.002
	ln PFNA	391	0.06	0.04; 0.08	**<0.0001**	385	0.05	0.03; 0.07	**<0.0001**	385	−0.02	−0.05; 0.01	0.194
	ln PFDA	391	0.08	0.05; 0.11	**<0.0001**	385	0.07	0.05; 0.09	**<0.0001**	385	0.004	−0.03; 0.04	0.850
	ln PFUnA	391	0.08	0.04; 0.12	**<0.0001**	385	0.06	0.03; 0.10	**0.001**	385	0.01	−0.05; 0.07	0.781
>29 years	LipPOPs (µg/kg lipid)												
	ln PCB153	718	−0.13	−0.15; −0.12	**<0.0001**	715	−0.13	−0.15; −0.12	**<0.0001**	715	−0.07	−0.08; −0.05	**<0.0001**
	ln *p,p’*-DDE	711	−0.13	−0.14; −0.12	**<0.0001**	708	−0.13	−0.14; −0.12	**<0.0001**	708	−0.06	−0.08; −0.05	**<0.0001**
	ln Oxychlordane	703	−0.15	−0.17; −0.14	**<0.0001**	700	−0.15	−0.17; −0.14	**<0.0001**	700	−0.08	−0.10; −0.06	**<0.0001**
	Metals (µg/L)												
	ln Hg	557	−0.08	−0.10; −0.07	**<0.0001**	554	−0.09	−0.10; 0.07	**<0.0001**	554	−0.03	−0.05; −0.02	**<0.0001**
	ln Pb	553	−0.10	−0.11; −0.09	**<0.0001**	550	−0.10	−0.11; −0.09	**<0.0001**	550	−0.07	−0.08; −0.06	**<0.0001**
	ln Se	555	−0.01	−0.01; 0.003	0.200	552	−0.01	−0.02; 0.002	0.162	552	−0.01	−0.004; 0.02	0.210
	PFASs (ng/mL)												
	ln PFHxS	381	−0.12	−0.13; −0.11	**<0.0001**	378	−0.12	−0.13; −0.11	**<0.0001**	378	−0.16	−0.20; −0.11	**<0.0001**
	ln PFOS	381	−0.09	−0.11; −0.08	**<0.0001**	378	−0.10	−0.11; −0.08	**<0.0001**	378	−0.13	−0.18; −0.08	**<0.0001**
	ln PFOA	381	−0.05	−0.07; −0.04	**<0.0001**	378	−0.06	−0.07; −0.04	**<0.0001**	378	−0.11	−0.16; −0.07	**<0.0001**
	ln PFNA	381	3.5e−5	−0.13; 0.13	0.996	378	−0.002	−0.02; 0.01	0.741	378	−0.009	−0.14; −0.04	**<0.0001**
	ln PFDA	381	0.02	−1.5e−4; 0.03	0.052 ^#^	378	0.01	−0.002; 0.03	0.096 ^#^	378	−0.05	−0.11; 0.01	0.086 ^#^
	ln PFUnA	381	0.01	−0.004; 0.03	0.133	378	0.01	−0.01; 0.03	0.236	378	−0.08	−0.15; −0.01	0.022

LipPOP: legacy lipophilic POPs; PFASs: perfluoroalkylated substances; ^a^ adjusted for district; ^b^ adjusted for district and pregnancy status (pregnant women: age < = 29 years: *n* = 537 for lipPOPs and metals; *n* = 372 for PFASs. Age > 29 years: n = 304 for lipPOP and metal; *n* = 211 for PFASs). *n*: number of participants in the analysis. Bold indicates statistical significance (*p* <0.05), ^#^: borderline significance (0.05 < *p* < 0.10). The Greenland women data were collected from the following districts: Qaanaaq (*n* = 37), Uummannaq (*n* = 16), Qeqertarsuaq (*n* = 45), Ilulissat (*n* = 408), Aasiaat (*n* = 29), Sisimiut (*n* = 109), Maniitsoq (*n* = 46), Nuuk (*n* = 720), Paamiut (*n* = 4), Narsaq (*n* = 42), Tasiilaq (*n* = 75) and Ittoqqortoormiit (*n* = 43). For the full name of chemicals, please refer to the abbreviation list.

**Table 7:** Women in Nuuk and Ilulissat: Changes in the levels of lipPOPs, metals and PFASs, given as the time to halve the concentration (T_1/2_) or the time to double the concentration (T_2_).

Chemical	*n*	Time Range (Year)	Raw			Adjusted ^a^			Adjusted ^b^		
			Change per Year	T_1/2,_−Years T_2_, +years	*p*	Change per Year	T_1/2_,−Years T_2_, +Years	*p*	Change per Year	T_1/2_,−yeas T_2_, +Years	*p*
Nuuk, women											
PCB153 (µg/kg lipid)	719	1999–2015	−12.5%	−5.21	**<0.0001**	−8.52%	−7.79	**<0.0001**	−8.61%	−7.70	**<0.0001**
*p,p’*-DDE (µg/kg lipid)	719	1999–2015	−12.5%	−5.21	**<0.0001**	−8.61%	−7.70	**<0.0001**	−8.61%	−7.70	**<0.0001**
Oxychlordane (µg/kg lipid)	719	1999–2015	−13.1%	−4.95	**<0.0001**	−8.61%	−7.70	**<0.0001**	−8.61%	−7.70	**<0.0001**
Total Hg (µg/L)	579	1999–2015	−1.00%	−69.3	0.129	−0.90%	−77.0	0.140	−1.98%	−34.7	**0.010**
Pb (µg/L)	579	1999–2015	−6.57%	−10.2	**<0.0001**	−6.48%	−10.4	**<0.0001**	−6.76%	−9.90	**<0.0001**
Se (µg/L)	578	1999–2015	−2.18%	−31.5	**<0.0001**	−2.18%	−31.5	**<0.0001**	−1.98%	−34.7	**<0.0001**
PFHxS (ng/mL)	491	1998–2015	−12.6%	−5.13	**<0.0001**	−11.7%	−5.59	**<0.0001**	−13.9%	−4.60	**<0.0001**
PFOS (ng/mL)	491	1998–2015	−10.3%	−6.36	**<0.0001**	−7.41%	−9.00	**<0.0001**	−5.82%	−11.6	**0.002**
PFOA (ng/mL)	491	1998–2015	−6.57%	−10.2	**<0.0001**	−6.67%	−10.1	**<0.0001**	−4.88%	−13.9	**<0.0001**
PFNA (ng/mL)	491	1998–2015	−0.90%	−77.0	0.074#	+0.20%	+347	0.845	+1.00%	+69.3	0.439
PFDA (ng/mL)	491	1998–2015	+0.10%	+693	0.863	+2.33%	+30.1	0.090#	+2.02%	+34.7	0.326
PFUnA (ng/mL)	491	1998–2015	+0.10%	+693	0.885	+3.56%	+19.8	0.058#	+3.05%	+23.1	0.338
Ilulissat, women											
PCB153 (µg/kg lipid)	347	1994–2015	−7.04%	−9.50	**<0.0001**	−6.85%	−9.76	**<0.0001**	−4.88%	−13.9	**<0.0001**
*p,p’*-DDE (µg/kg lipid)	330	1994–2015	−6.76%	−9.90	**<0.0001**	−6.67%	−10.1	**<0.0001**	−6.76%	−9.9	**<0.0001**
Oxychlordane (µg/kg lipid)	347	1994–2015	−6.29%	−10.7	**<0.0001**	−6.11%	−11.0	**<0.0001**	−4.88%	−13.9	**<0.0001**
Total Hg (µg/L)	299	1994–2015	−6.01%	−11.2	**<0.0001**	−5.92%	−11.4	**<0.0001**	−0.7%	−99.0	0,679
Pb (µg/L)	300	1994–2015	−9.43%	−7.00	**<0.0001**	−9.43%	−7.00	**<0.0001**	−1.98%	−34.7	**0.020**
Se (µg/L)	296	1994–2015	+4.71%	+15.1	**<0.0001**	+4.71%	+15.1	**<0.0001**	+4.08%	+17.3	**<0.0001**

LipPOP: legacy lipophilic POPs; PFASs: perfluoroalkylated substances; ^a^ adjusted for age; ^b^ adjusted for age and pregnancy status (Nuuk pregnant women: *n* = 554 for lipPOP and metals, *n* = 354 for PFASs; Ilulissat pregnant women: *n* = 111 for lipPOP and metals). Bold indicate statistical significance (*p* <0.05). ^#^: Borderline significant (0.05 < *p* < 0.10). *n*: number of participants in the analysis. PFASs in Ilulissat participants were not performed time trend analysis stratified by age due to small sample size (*n* = 79) and only had four time series. T_1/2_: half-time, T_2_: doubling time. For the full name of chemicals, please refer to the abbreviation list.

**Table 8:** Women and men across Greenlandic: changes in the levels of lipPOPs, metals and PFASs, given as the time to halve the concentration (T_1/2_) or the time to double the concentration (T_2_).

Chemical	*n*	Time Range (Year)	Raw			Adjusted ^a^			Adjusted ^b^		
			Change per Year	T_1/2,_ -Years; T_2_, +Years	*p*	Change per Year	T_1/2_, -Years; T_2_, +Years	*p*	Change per Year	T_1/2_, -Years; T_2_, +Years	*p*
Greenland, women^¤^											
PCB153 (µg/kg lipid)	1522	1994–2015	−9.52%	−6.93	**<0.0001**	−8.61%	−7.70	**<0.0001**	−5.82%	−11.6	**<0.0001**
*p,p’*-DDE (µg/kg lipid)	1504	1994–2015	−9.52%	−6.93	**<0.0001**	−8.61%	−7.70	**<0.0001**	−5.82%	−11.6	**<0.0001**
Oxychlordane (µg/kg lipid)	1506	1994–2015	−10.4%	−6.30	**<0.0001**	−9.52%	−6.93	**<0.0001**	−5.82%	−11.6	**<0.0001**
Total Hg (µg/L)	1315	1994–2015	−6.76%	−9.90	**<0.0001**	−6.76%	−9.90	**<0.0001**	−1.98%	−34.7	**<0.0001**
Pb (µg/L)	1311	1994–2015	−8.61%	−7.70	**<0.0001**	−8.61%	−7.70	**<0.0001**	−5.82%	−11.6	**<0.0001**
Se (µg/L)	1312	1994–2015	+1.01%	+69.3	**<0.0001**	+1.01%	+69.3	**0.001**	+1.01%	+69.3	**0.001**
PFHxS (ng/mL)	792	1997–2015	−11.3%	−5.78	**<0.0001**	−10.4%	−6.30	**<0.0001**	−14.8%	−4.33	**<0.0001**
PFOS (ng/mL)	792	1997–2015	−8.61%	−7.70	**<0.0001**	−5.82%	−11.6	**<0.0001**	−8.61%	−7.70	**<0.0001**
PFOA (ng/mL)	792	1997–2015	−3.92%	−17.3	**<0.0001**	−7.69%	−8.66	**<0.0001**	−7.69%	−8.66	**<0.0001**
PFNA (ng/mL)	792	1997–2015	+2.02%	+34.7	**0.001**	+3.05%	+23.1	**<0.0001**	−4.88%	−13.9	**0.001**
PFDA (ng/mL)	792	1997–2015	+3.05%	+23.1	**<0.0001**	+5.13%	+13.9	**<0.0001**	−1.98%	−34.7	0.364
PFUnA (ng/mL)	792	1997–2015	+3.05%	+23.1	**<0.0001**	+5.13%	+13.9	**<0.0001**	−2.96%	−23.1	0.250
Greenland, men ^¤¤^											
PCB153 (µg/kg lipid)	400	1997–2006	−8.24%	−8.06	**<0.0001**	−11.3%	−5.80	**<0.0001**			
*p,p’*-DDE (µg/kg lipid)	400	1997–2006	−4.50%	−15.1	**0.001**	−8.61%	−7.70	**<0.0001**			
Oxychlordane (µg/kg lipid)	386	1997–2006	−10.3%	−6.36	**<0.0001**	−15.6%	−4.10	**<0.0001**			
Total Hg (µg/L)	349	1999–2006	−4.30%	−15.8	**0.018**	−13.1%	−5.00	**<0.0001**			
Pb (µg/L)	346	1999–2006	−9.43%	−7.00	**<0.0001**	−12.2%	−5.30	**<0.0001**			
Se (µg/L)	350	1999–2006	+1.51%	+46.2	0.289	−4.88%	−13.9	0.343			
PFHxS (ng/mL)	74	1997–2006	−11.2%	−5.82	**<0.0001**	−9.52%	−6.90	**<0.0001**			
PFOS (ng/mL)	74	1997–2006	−13.0%	−4.99	**<0.0001**	−9.52%	−6.90	**<0.0001**			
PFOA (ng/mL)	74	1997–2006	−12.5%	−5.21	**<0.0001**	−5.82%	−11.6	**<0.0001**			
PFNA (ng/mL)	74	1997–2006	−6.76%	−9.90	**0.035**	−5.82%	−11.6	**0.010**			
PFDA (ng/mL)	74	1997–2006	−4.40%	−15.4	0.272	+1.01%	+69.3	0.241			
PFUnA (ng/mL)	74	1997–2006	−4.40%	−15.4	0.275	−9.52%	−6.90	0.262			

LipPOP: legacy lipophilic POPs; PFASs: perfluoroalkylated substances; ^a^ adjusted for age and district. ^b^ adjusted for age, district and pregnancy status (pregnant women: n = 842 for lipPOPs and metals, n = 583 for PFASs). *n*: number of participants in the analysis. Bold indicate statistical significance (*p* <0.05). ^#^: borderline significance (0.05 < *p* < 0.10). ^¤:^ The Greenland women data were collected from the following districts: Qaanaaq (n = 37), Uummannaq (*n* = 16), Qeqertarsuaq (*n* = 45), Ilulissat (*n* = 408), Aasiaat (*n* = 29), Sisimiut (*n* = 109), Maniitsoq (*n* = 46), Nuuk (*n* = 720), Paamiut (*n* = 4), Narsaq (*n* = 42), Tasiilaq (*n* = 75) and Ittoqqortoormiit (*n* = 43); ^¤¤:^ The Greenland men data were collected from the following districts: Qaanaaq (*n* = 41), Upernavik (*n* = 11), Uummannaq (*n* = 62), Qeqertarsuaq (*n* = 35), Ilulissat (*n* = 14 ), Sisimiut (*n* = 51), Nuuk (*n* = 49), Narsaq (*n* = 30), Tasiilaq (*n* = 40) and Ittoqqortoormiit (*n* = 67). T_1/2_: half-time, T_2_: doubling time. For the full name of chemicals, please refer to the abbreviation list.

## Data Availability

Not applicable.

## Supplementary Materials

The following are available online at https://www.mdpi.com/1660-4601/18/5/2774/s1: Table S1: The time series of the blood levels of POPs and metals in Greenland population. Table S2: Greenlandic Men: age stratified time trend of lipPOPs and heavy metal level during 1997–2006. Table S3: Women and Men across Greenland: Time trend of lipPOPs, metals and PFASs level during 1994–2015. Table S4: Levels of lipPOPs and metals in pregnant and nonpregnant women. Table S5: Greenland women: Time trend of lipPOPs, metals and PFASs levels stratified by the pregnancy status. Figure S1. Time trend of lip POPs, metals and PFASs in Nuuk women during 1998-2015. Figure S2. Time trend of lip POPs and metals in Ilulissat women during 1994-2015. Figure S3. The levels of PFASs in Ilulissat women in 1999 (n = 10), 2011 (n = 33), 2014 (n = 20) and 2015 (n = 6).

## Abbreviations

AA	arachidonic acid C20:4n6
ACCEPT	Adapting to Climate Change, Environmental Pollution and Dietary Transition
AMAP	Arctic Monitoring and Assessment Programme
ANCOVA	Analysis of covariance
BMI	body mass index
DDT	2,2-bis(p-chlorophenyl)−1,1,1-trichloroethane
deca-BDE	decabromodiphenyl ether
DHA	docosahexaenoic acid C22:6n3
EPA	eicosapentaenoic acid C20:5n3
HBCDD	hexabromocyclodecane
HCB	hexachlorobenzene
Hg	mercury
ICP–MS	Inductively Coupled Plasma Mass Spectrometry
LipPOP	lipophilic POP
OCPs	organchlorine pesticides
n−3/n−6	n−3 polyunsaturated fatty acids/n−6 polyunsaturated fatty acids
Pb	lead
PBDEs	polybrominated diphenyl ethers
PCBs	polychlorinated biphenyls
PCDD/PCDF	polychlorinated dibenzo-p-dioxins/furans
PFASs	perfluoroalkylated substances
PFBS	perfluorobutanesulfonic acid
PFCAs	perfluorinated carboxylic acids
PFDA	perfluorodecanoic acid
PFDoA	perfluorododecanoic acid
PFDS	perfluorodecanesulfonate
PFHpA	perfluoroheptanoic acid
PFHpS	perfluoroheptanesulfonate
PFHxA	perfluorohexanoic acid
PFHxS	perfluorohexane sulfonate
PFHxSF	perfluorohexane sulfonyl fluoride
PFOS	perfluorooctanoic acid
PFOA	perfluorooctane sulfonate
PFOSA	perfluorooctane sulfonamide
PFNA	perfluorononanoic acid
PFPeA	perfluoropentanoic acid
PFSAs	perfluorinated sulfonic acids
PFTeA	perfluorotetradecanoic acid
PFTrA	perfluorotridecanoic acid
PFUnA	perfluoroundecanoic acid
POPs	persistent organic pollutants
p,p’-DDE	1,1-dichloro−2,2-bis (p-chlorophenyl)-ethylene
S	supplementary
Se	selenium
