# Supplementary material for: Time Trend of Persistent Organic Pollutants and Metals in Greenlandic Inuit during 1994–2015
Source: Int J Environ Res Public Health. 2021 Mar 9;18(5):2774. doi: 10.3390/ijerph18052774 (PMC7967253; doi:10.3390/ijerph18052774)
Supplement: Supplementary file 1 [file ijerph-18-02774-s001.pdf]

Table S1. The time series of the blood levels of POPs and metals in Greenland population

Year	Nuuk						Ilulissat						All Greenlandic districts*					
	Women (n)			Men (n)			Women (n)			Men (n)			Women (n)			Men (n)		
	lipPOP	Metals	PFAS	lipPOP	Metals	PFAS	lipPOP	Metals	PFAS	lipPOP	Metals	PFAS	lipPOP	Metals	PFAS	lipPOP	Metals	PFAS
1994							12 (0)	14 (0)					12 (0)	14 (0)				
1995							111 (0)	111 (0)					111 (0)	111 (0)				
1996							72 (0)	32 (0)					72 (0)	32 (0)				
1997				11						14		9	24 (0)		2 (0)	51		25
1998			10 (0)												10 (0)			
1999	14 (14)	13 (13)					27 (0)	28 (0)	10 (0)				83 (14)	84 (13)	10 (0)	100	100	
2000	87 (31)	22 (22)	56 (0)										135 (31)	70 (22)	63 (0)	40	40	3
2001	44 (44)	35 (35)											44 (44)	35 (35)	7 (0)			2
2002	107 (41)	39 (29)	66 (0)										149 (41)	81 (39)	66 (0)	51	50	
2003	30 (30)	30 (30)					9 (9)	9 (9)					74 (40)	76 (40)	16 (0)	41	43	20
2004	29 (29)	29 (29)					22 (22)	22 (22)					73 (58)	60 (60)	10 (0)			9
2005	56 (12)	57 (12)	5 (0)	38	50	5	25 (25)	25 (25)					82 (38)	83 (38)	5 (0)	38	50	5
2006													87 (0)	87 (0)	20 (0)	65	66	10
2010	40 (40)	41 (41)	41 (41)										56 (56)	57 (57)	57 (57)			
2011	50 (50)	52 (52)	53 (53)				29 (29)	33 (33)	33 (33)				123 (123)	132 (132)	133 (133)			
2013	80 (80)	77 (77)	77 (77)										90 (90)	87 (87)	87 (87)			
2014	124 (124)	124 (124)	124 (124)				20 (20)	19 (19)	20 (20)				210 (210)	209 (209)	209 (209)			
2015	59 (59)	59 (59)	59 (59)				6 (6)	6 (6)	6 (6)				97 (97)	97 (97)	97 (97)			

*All Greenland districts include Qaanaaq, Upernavik, Uummannaq, Qeqertarsuaq, Ilulissat, Aasiaat, Sisimiut, Maniitsoq, Nuuk, Paamiut, Narsaq, Tasiilaq, and Ittoqqortoormiit. The values in the bracket are numbers of pregnant women. lipPOPs include polychlorinated biphenyls (PCBs) and organochlorine pesticides (OCPs) regulated on the Stockholm Convention list. PFAS: perfluoroalkylated substances. n: number of participants with information

Table S2. Greenlandic Men: age stratified time trend of lipPOPs and heavy metal level during 1997-2006.

**Men (1997-2006),** Median age 37 years (min-max:18-73)																
	<=37 years								>37 years							
	Raw				Adjusted[a]				Raw				Adjusted[a]			
	n	β	95% CI	p	n	β	95% CI	p	n	β	95% CI	p	n	β	95% CI	p
**LipPOPs (μg/kg lipid)**																
ln PCB153	217	-0.13	-0.17; -0.09	**<0.0001**	217	-0.13	-0.17; -0.08	**<0.0001**	183	-0.11	-0.14; -0.07	**<0.0001**	183	-0.10	-0.14; -0.06	**<0.0001**
ln p,p'-DDE	217	-0.09	-0.13; -0.05	**<0.0001**	217	-0.10	-0.14; -0.06	**<0.0001**	183	-0.06	-0.09; -0.02	**0.001**	183	-0.06	-0.09; -0.02	**0.001**
lnOxychlordane	216	-0.15	-0.20; -0.10	**<0.0001**	216	-0.17	-0.22; -0.12	**<0.0001**	170	-0.14	-0.18; -0.10	**<0.0001**	170	-0.15	-0.19; -0.10	**<0.0001**
**Metals (μg/L)**																
ln Hg	184	-0.02	-0.09; 0.04	0.453	184	-0.12	-0.18; -0.06	**<0.0001**	165	-0.10	-0.14; -0.06	**<0.0001**	165	-0.12	-0.16; -0.09	**<0.0001**
ln Pb	183	-0.14	-0.17; -0.10	**<0.0001**	183	-0.14	-0.17; -0.10	**<0.0001**	163	-0.10	-0.14; -0.07	**<0.0001**	163	-0.10	-0.14; -0.07	**<0.0001**
ln Se	184	0.03	-0.01; 0.07	0.149	184	-0.03	-0.07; 0.01	0.146	166	-0.03	-0.06; 0.01	0.210	166	-0.05	-0.08; -0.11	**0.009**

[a]data were adjusted for district; The men data were collected from the following districts: Qaanaaq (n=41), Upernavik (n=11), Uummannaq (n=62), Qeqertarsuaq (n=35), Ilulissat(n=14), Sisimiut (n=51), Nuuk (n=49), Narsaq (n=30), Tasiilaq (n=40), Ittoqqortoormiit (n=67). n: number of participants in the analysis. Bold indicate statistical significance (p <0.05). The age stratified time trend for PFASs was not carried out due to small sample size (n=74)

Table S3. Women and Men across Greenland: Time trend of lipPOPs, metals and PFASs level during 1994-2015

Women+Men (1994-2015), Median age 30 years (min-max:15-79)

	Raw				Adjusted [a]			
	n	β	95% CI	*p*	n	95% CI	β	*p*
**LipPOPs (μg/kg lipid)**								
ln PCB153	1926	-0.12	-0.13; -0.11	**<0.0001**	1912	-0.09	-0.10; -0.08	**<0.0001**
ln p,p'-DDE	1908	-0.11	-0.12; -0.10	**<0.0001**	1894	-0.08	-0.09; -0.08	**<0.0001**
ln Oxychlordane	1896	-0.13	-0.14; -0.12	**<0.0001**	1882	-0.10	-0.11; -0.09	**<0.0001**
**Metals (μg/L)**								
lnHg	1668	-0.09	-0.10; 0.08	**<0.0001**	1653	-0.07	-0.08; -0.06	**<0.0001**
lnPb	1661	-0.11	-0.11; -0.10	**<0.0001**	1646	-0.10	-0.10; -0.09	**<0.0001**
lnSe	1666	-0.001	0.006; 0.004	0.663	1651	0.01	0.04; 0.01	**<0.0001**

Women+Men (1997-2015), Median age 30 years (min-max:16-73)

	Raw				Adjusted [a]			
	n	β	95% CI	p	n	β	95% CI	p
**PFASs (ng/mL)**								
ln PFHxS	867	-0.12	-0.13; -0.12	**<0.0001**	836	-0.11	-0.12; -0.09	**<0.0001**
ln PFOS	867	-0.08	-0.09; -0.08	**<0.0001**	836	-0.06	-0.07; -0.04	**<0.0001**
ln PFOA	867	-0.04	-0.05; -0.03	**<0.0001**	836	-0.02	-0.03; -0.01	**0.003**
ln PFNA	867	0.02	0.01; 0.03	**<0.0001**	836	0.04	0.02; 0.05	**<0.0001**
ln PFDA	867	0.03	0.02; 0.04	**<0.0001**	836	0.06	0.05; 0.08	**<0.0001**
ln PFUnA	867	0.04	0.03; 0.05	**<0.0001**	836	0.06	0.04; 0.08	**<0.0001**

[a] adjusted for age, sex and district; The data were collected from the following districts: Qaanaaq(n=78), Upernavik(n=11), Uummannaq(n=78), Qeqertarsuaq(n=80), Ilulissat(n=374), Aasiaat(n=29), Sisimiut(n=160), Maniitsoq(n=46), Nuuk(n=769), Paamiut(n=4), Narsaq(n=72), Tasiilaq(n=115), Ittoqqortoormiit(n=110). n: number of participants in the analysis. Bold indicate statistical significance (p <0.05).

Table S4. Levels of lipPOPs (μg/kg lipid) and metals (μg/L) in pregnant and non-pregnant women

		Greenland women					Nuuk women				
		Pregnant women		Non-pregnant women		*p*	Pregnant women		Non-pregnant women		*p* ¤
Sampling year		n	**Median** (mix-max)	n	**Median** (mix-max)		n	**Median** (mix-max)	n	**Median** (mix-max)	
1999	*Age, years*	*14*	*26 (19-37)*	*71*	*32 (16-45)*						
	PCB153	14	108 (59.1-425)	69	612 (43.4-4871)	**0.0010***					
	p,p'-DDE	14	248 (100-1023)	69	854 (74.5-5847)	**0.0030***					
	Oxychlordane	14	22.1 (6.65-98.3)	69	168 (1.92-2132)	**<0.0001***					
	Total Hg	13	3.07 (0.50-14.2)	71	17.1 (1.74-78.2)	**<0.0001***					
	Pb	13	32.0 (25.4-56.9)	71	51.2 (24.0-156)	**0.0050***					
	Se	13	100 (65.1-584)	71	209 (83.8-817)	0.4100*					
2000	*Age, years*	*32*	*27 (19-37)*	*104*	*50 (19-64)*		*32*	*27 (16-45)*	*56*	*56 (50-64)*	
	PCB153	31	117 (28.6-595)	104	538 (31.3-1960)	0.290*	31	117 (28.6-595)	56	574 (93.8-1759)	**<0.0001**
	p,p'-DDE	31	279 (59.0-1206)	104	1220 (67.5-6464)	0.080*#	31	279 (59.0-1206)	56	1241 (167-6464)	**<0.0001**
	Oxychlordane	31	33.2 (1.93-154)	104	209 (12.7-722)	0.1900*	31	33.2 (1.93-154)	56	216 (15.9-722)	**<0.0001**
	Total Hg	22 (Nuuk)	3.80 (0.50-14.0)	48 (Tasiilaq)	27.4 (6.50-155)	**<0.0001¤**	-	-	-	-	
	Pb	22 (Nuuk)	29.9 (6.22-75.8)	48 (Tasiilaq)	33.0 (16.0-141)	**0.040¤**	-	-	-	-	
	Se	22 (Nuuk)	131 (58.9-424)	48 (Tasiilaq)	173 (90.5-415)	**0.020¤**	-	-	-	-	
2002	*Age, years*	*42*	*28 (15-43)*	*108*	*52 (18-66)*		*42*	*28 (15-43)*	*66*	*58 (50-66)*	
	PCB153	41	130 (28.5-910)	108	426 (20.2-1759)	0.970*	41	130 (28.5-910)	66	603 (194-1759)	**<0.0001**
	p,p'-DDE	41	313 (45.3-2300)	108	865 (37.0-6464)	0.320*	41	312 (45.3-2300)	66	1277 (320-6464)	**<0.0001**
	Oxychlordane	41	39.5 (1.76-305)	108	138 (2.07-722)	0.760*	41	39.5 (1.76-305)	66	218 (31.3-722)	**<0.0001**
	Total Hg	39 (Nuuk)	4.61 (0.80-22.0)	42 (Sisimiut)	8.90 (1.20-33.2)	**0.003¤**	-	-	-	-	
	Pb	39 (Nuuk)	13.0 (5.18-80.8)	42 (Sisimiut)	30.0 (8.00-118)	**<0.0001¤**	-	-	-	-	
	Se	39 (Nuuk)	167 (110-576)	42 (Sisimiut)	169 (69.0-363)	**0.020¤**	-	-	-	-	
2003	*Age, years*	*40*	*28 (19-44)*	*36*	*35 (18-44)*						
	PCB153	40	98.5 (20.0-1200)	34	440 (30.2-2350)	**0.007***					
	p,p'-DDE	40	210 (34.0-2700)	34	683 (27.6-3296)	**0.030***					
	Oxychlordane	40	24.0 (1.70-500)	34	158 (5.37-1249)	0.070*					
	Total Hg	40	3.65 (0.41-16.0)	36	52.2 (5.70-164)	0.060*#					
	Pb	40	11.0 (4.50-71.0)	31	32.0 (18.0-164)	0.470*					
	Se	40	120 (74.0-550)	36	568 (120-1910)	**0.001***					
2004	*Age, years*	*65*	*24 (16-39)*	*15*	*46 (31-62)*						
	PCB153	58	90.0 (23.0-660)	15	457 (152-1068)	**0.040***					
	p,p'-DDE	58	195 (40.0-970)	15	1000 (234-2985)	0.080*#					
	Oxychlordane	58	30.5 (2.80-290)	-	-						
	Total Hg	60	3.85 (0.17-39.0)	-	-						
	Pb	60	8.75 (3.30-42.0)	-	-						
	Se	60	130 (80.0-1400)	-	-						
2005	*Age, years*	*37*	*25 (17-41)*	*45*	*38 (19-45)*		*12*	*27 (19-36)*	*45*	*38 (19-45)*	
	PCB153	38	92.5 (15.0-760)	44	108 (32.7-927)	0.340*	12	87.5 (25-400)	44	108 (32.7-926)	0.829
	p,p'-DDE	38	210 (31.0-1618)	44	296 (50.9-1618)	0.740*	12	170 (43-690)	44	296 (50.9-1618)	0.735
	Oxychlordane	38	35.0 (3.30-370)	43	23.2 (3.96-250)	0.060*#	12	32.0 (3.3-120)	43	23.2 (3.96-250)	0.486
	Total Hg	38	4.70 (0.67-20.0)	45	1.72 (0.48-9.89)	**0.050***	-	-	-	-	
	Pb	38	11.5 (5.90-57.0)	45	16.1 (5.09-101)	0.750*	-	-	-	-	
	Se	38	120 (84.0-420)	45	112 (57.9-428)	**0.050***	-	-	-	-	
All ❃ (1994-2015)	*Age, years*	864	27 (15-48)	766	32 (15-70)						
	PCB153	842	62.0 (5.10-4871)	741	271 (20.2-4871)	**<0.0001***					
	p,p'-DDE	841	140 (5.00-8800)	717	579 (27.6-6464)	**<0.0001***					
	Oxychlordane	842	19.0 (0.25-920)	724	86.7 (1.06-2132)	**<0.0001***					
	Total Hg	829	3.92 (0.17-73.0)	506	13.7 (0.48-164)	**<0.0001***					
	Pb	829	8.32 (1.05-80.8)	502	30.6 (2.09-404)	**<0.0001***					
	Se	829	123 (45.4-2795)	502	119 (3.80-1910)	**0.003***					

* The comparison was adjusted for age and district; ¤ comparison was adjusted only for age due to only one district had data. Bold indicate statistical significance (p <0.05), #: borderline significance (0.05<p<0.10). n: number of participants in the analysis, ❃: 2010-2015 only pregnant women see Suppl Table 5. -: no data available. No comparison was performed for PFASs since there was no data of same year for pregnant women and non-pregnant women

Table S5. Greenland women: Time trend of lipPOPs, metals and PFASs levels stratified by the pregnancy status

	**Pregnant women (1999-2015)** Median age 27 years (min-max: 15-48)								**Non-pregnant Women (1994-2006)** Median age 32 years (min-max: 15-70)							
	Raw				Adjusted [a]				Raw				Adjusted [a]			
	n	β	95% CI	*p*	n	β	95% CI	*p*	n	β	95% CI	*p*	n	β	95% CI	*p*
**LipPOPs (µg/kg lipid)**																
ln PCB153	842	-0.07	-0.08; -0.05	**<0.0001**	832	-0.07	-0.08; -0.06	**<0.0001**	680	0.12	-0.01; 0.03	0.261	680	-0.05	-0.06; -0.03	**<0.0001**
ln p,p'-DDE	841	-0.07	-0.08; 0.05	**<0.0001**	831	-0.07	-0.08; -0.06	**<0.0001**	663	0.01	-0.01; 0.03	0.319	663	-0.04	-0.06; -0.03	**<0.0001**
lnOxychlordane	842	-0.06	-0.08; -0.04	**<0.0001**	832	-0.06	-0.08; -0.05	**<0.0001**	664	-0.01	-0.04; 0.02	0.418	664	-0.07	-0.10; -0.05	**<0.0001**
**Metals (µg/L)**																
lnHg	829	-0.01	-0.02; 0.001	0.066#	819	-0.01	-0.02; 0.001	0.088#	486	-0.03	-0.05; -0.01	**0.012**	485	-0.04	-0.07; -0.02	**<0.0001**
lnPb	829	-0.06	-0.07; -0.05	**<0.0001**	819	-0.06	-0.07; -0.05	**<0.0001**	482	-0.07	-0.08; -0.05	**<0.0001**	481	-0.08	-0.10; -0.07	**<0.0001**
lnSe	829	-0.02	-0.02; -0.01	**<0.0001**	819	-0.02	-0.02; -0.01	**<0.0001**	483	0.10	0.08; 0.11	**<0.0001**	482	0.08	0.07; 0.10	**<0.0001**
	**Pregnant women (2010-2015)** Median age 27 years (min-max: 16-48)								**Non pregnant women (1997-2006)** Median age 53 years (min-max:18-66)							
**PFASs (ng/mL)**																
ln PFHxS	583	-0.15	-0.18; -0.13	**<0.0001**	574	-0.16	-0.19; -0.13	**<0.0001**	209	-0.21	-0.26; -0.16	**<0.0001**	189	-0.11	-0.19; -0.03	**0.008**
ln PFOS	583	-0.08	-0.11; -0.05	**<0.0001**	574	-0.08	-0.11; -0.05	**<0.0001**	209	-0.22	-0.28; -0.16	**<0.0001**	189	-0.07	-0.16; 0.01	0.082#
ln PFOA	583	-0.06	-0.09; -0.04	**<0.0001**	574	-0.06	-0.09; -0.03	**<0.0001**	209	-0.25	-0.31; -0.20	**<0.0001**	189	-0.07	-0.14; 0.00	**0.050**
ln PFNA	583	-0.05	-0.08; -0.02	**<0.0001**	574	-0.10	-0.07; -0.02	**<0.0001**	209	-0.07	-0.14; -0.01	**0.026**	189	0.001	-0.08; 0.09	0.974
ln PFDA	583	-0.03	-0.06; 0.01	0.112	574	-0.02	-0.05; 0.01	0.205	209	-0.08	-0.16; -0.01	**0.025**	189	0.08	-0.02; 0.18	0.115
ln PFUnA	583	-0.04	-0.09; 0.01	0.132	574	-0.03	-0.08; 0.02	0.179	209	-0.06	-0.14; 0.02	0.118	189	0.07	-0.04; 0.17	0.204

[a] adjusted for age and district; Bold indicate statistical significance (p <0.05), #: borderline significance (0.05<p<0.10). n: number of participants in the analysis

The pregnant women data were collected from the following districts: Qaanaaq (n=3), Uummannaq (n=1), Ilulissat (n=116), Aasiaat (n=31), Sisimiut (n=67), Maniitsoq (n=50), Nuuk (n=566), Paamiut (n=4), Tasiilaq (n=17), Ittoqqortoormiit (n=1).

The non-pregnant women data were collected from the following districts: Qaanaaq (n=34), Uummannaq (n=15), Qeqertarsuaq (n=45), Ilulissat (n=250), Sisimiut (n=42), Nuuk (n=166), Narsaq (n=42), Tasiilaq (n=58), Ittoqqortoormiit (n=42).

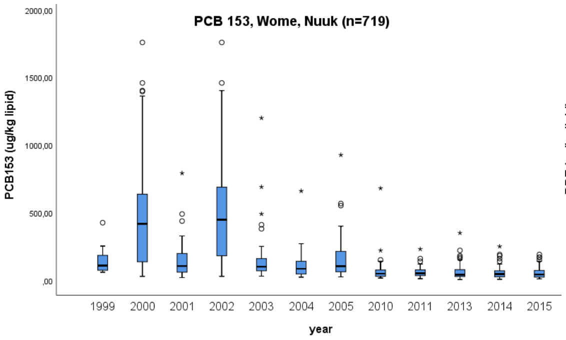
PCB 153, Wome, Nuuk (n=719)
PCB153 (ug/kg lipid)
1999 2000 2001 2002 2003 2004 2005 2010 2011 2013 2014 2015
year

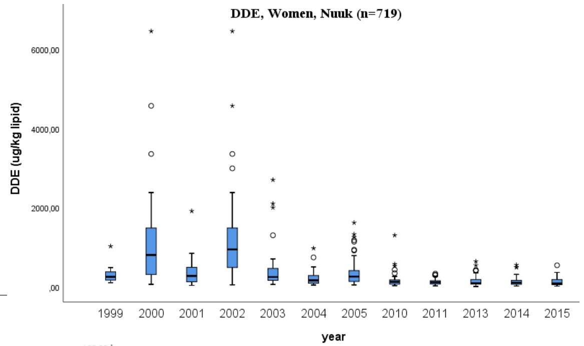
DDE, Women, Nuuk (n=719)
DDE (ug/kg lipid)
1999 2000 2001 2002 2003 2004 2005 2010 2011 2013 2014 2015
year

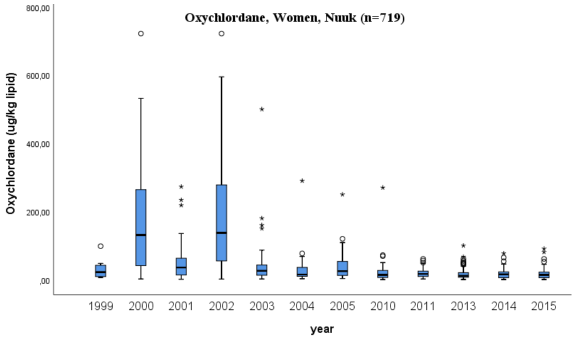
Oxychlordane, Women, Nuuk (n=719)
Oxychlordane (ug/kg lipid)
1999 2000 2001 2002 2003 2004 2005 2010 2011 2013 2014 2015
year

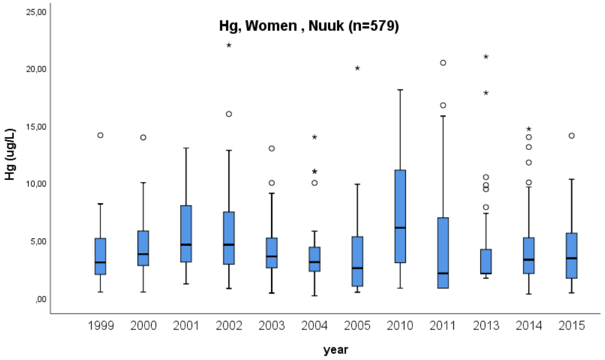
Hg, Women , Nuuk (n=579)
Hg (ug/L)
1999 2000 2001 2002 2003 2004 2005 2010 2011 2013 2014 2015
year

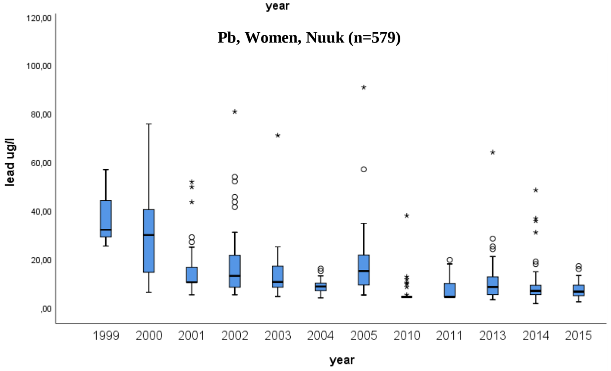
Pb, Women, Nuuk (n=579)
lead ug/l
1999 2000 2001 2002 2003 2004 2005 2010 2011 2013 2014 2015
year

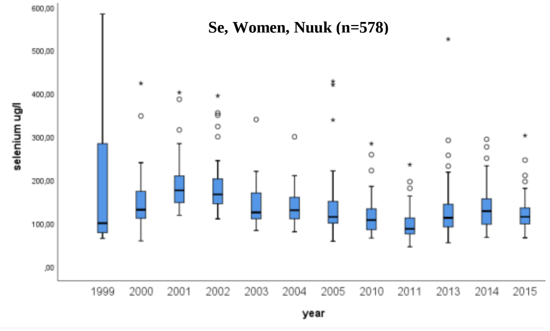
Se, Women, Nuuk (n=578)
selenium ug/l
1999 2000 2001 2002 2003 2004 2005 2010 2011 2013 2014 2015
year

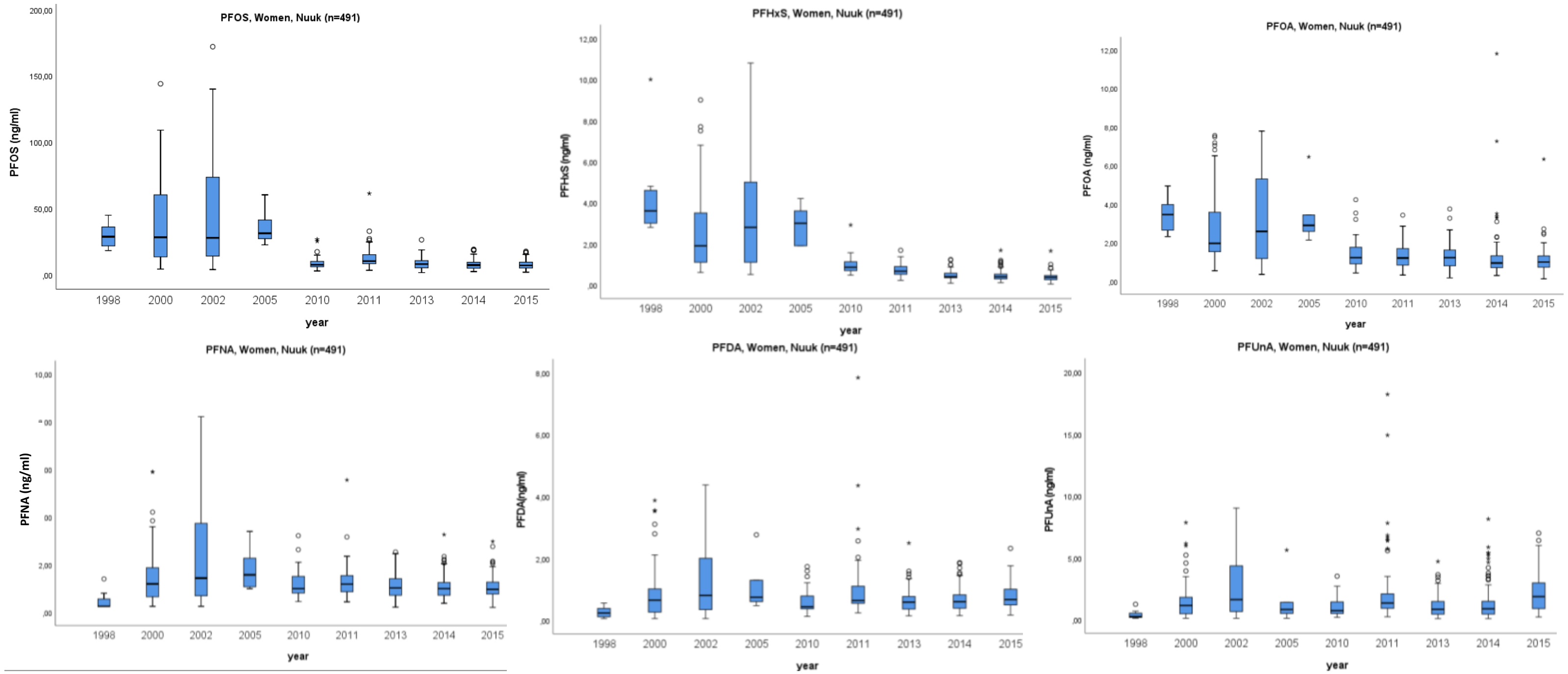

Figure S1. Time trend of lip POPs, metals and PFASs in Nuuk women during 1998-2015. The boxes display the 25th and 75th centiles, and the line inside the boxes represents the median value. The whiskers display the lower and upper values within 1.5 times the interquartile range beyond the box. Outliers are marked with dots. The data are raw data.

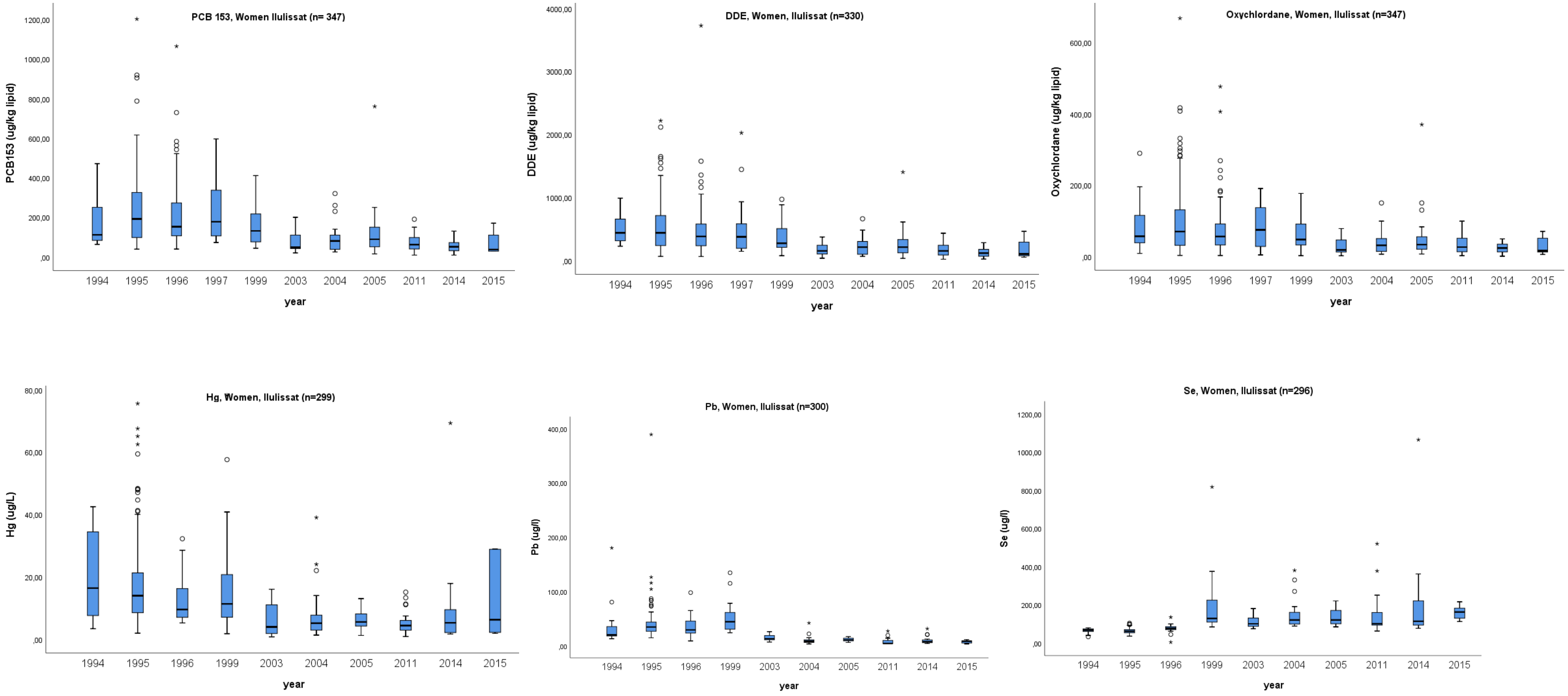

Figure S2. Time trend of lip POPs and metals in Ilulissat women during 1994-2015. The boxes display the 25th and 75th centiles, and the line inside the boxes represents the median value. The whiskers display the lower and upper values within 1.5 times the interquartile range beyond the box. Outliers are marked with dots. The data are raw data.

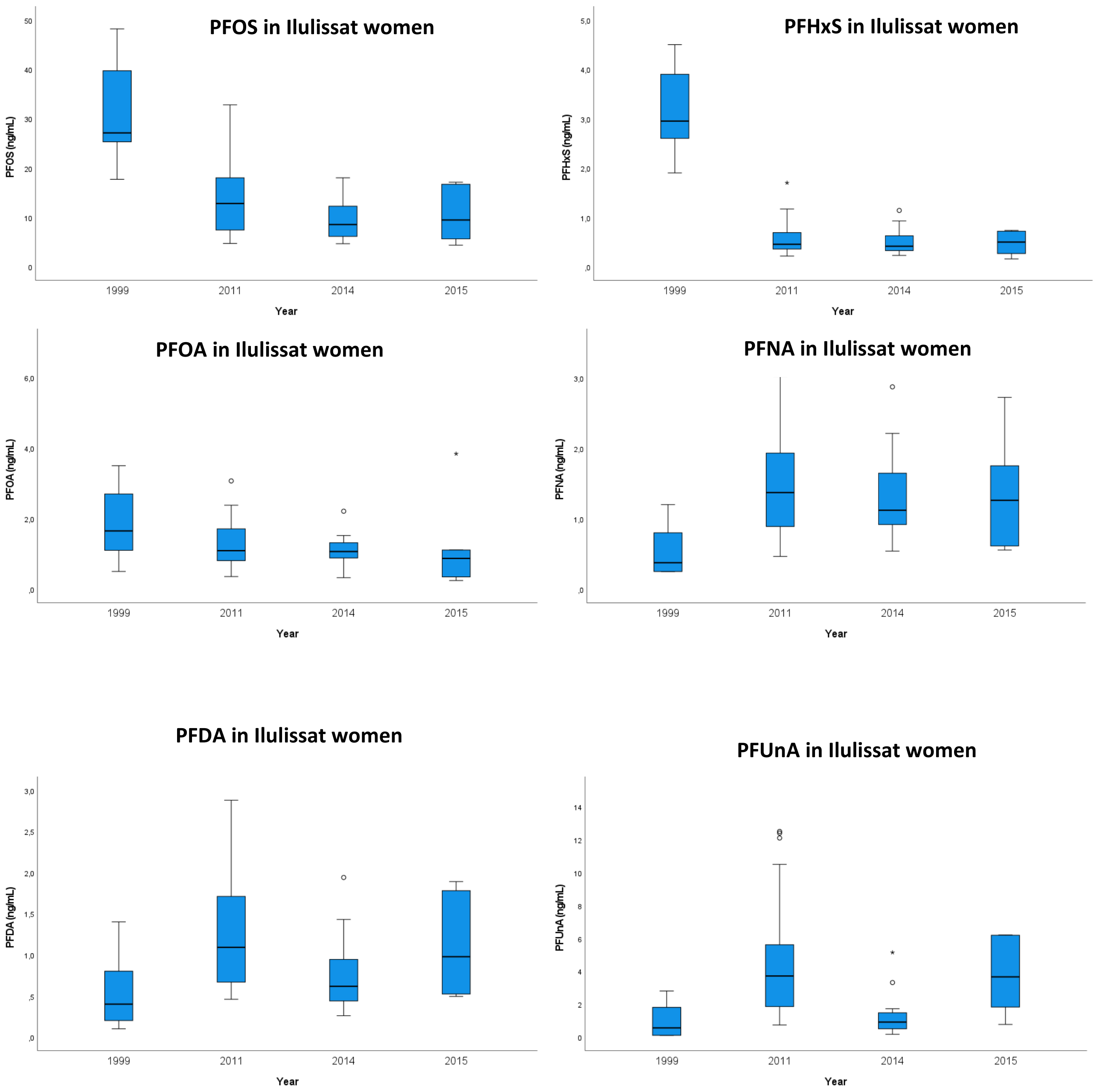

Figure S3. The levels of PFASs in Ilulissat women in 1999 (n=10), 2011(n=33), 2014 (n=20) and 2015 (n=6). The boxes display the 25th and 75th centiles, and the line inside the boxes represents the median value. The whiskers display the lower and upper values within 1.5 times the interquartile range beyond the box. The data are raw data.
